# A 3D‐Printed Dual Driving Forces Scaffold with Self‐Promoted Cell Absorption for Spinal Cord Injury Repair

**DOI:** 10.1002/advs.202301639

**Published:** 2023-10-23

**Authors:** Chen Qiu, Yuan Sun, Jinying Li, Jiayi Zhou, Yuchen Xu, Cong Qiu, Kang Yu, Jia Liu, Yuanqing Jiang, Wenyu Cui, Guanghao Wang, He Liu, Weixin Yuan, Tuoying Jiang, Yaohui Kou, Zhen Ge, Zhiying He, Shaomin Zhang, Yong He, Luyang Yu

**Affiliations:** ^1^ Key Laboratory of Cardiovascular Intervention and Regenerative Medicine of Zhejiang Province Department of Cardiology Sir Run Run Shaw Hospital Zhejiang University Hangzhou 310058 China; ^2^ MOE Laboratory of Biosystems Homeostasis & Protection and iCell Biotechnology Regenerative Biomedicine Laboratory of College of Life Sciences Zhejiang University Hangzhou 310058 China; ^3^ State Key Laboratory of Fluid Power and Mechatronic Systems School of Mechanical Engineering Zhejiang University Hangzhou 310027 China; ^4^ Key Laboratory of 3D Printing Process and Equipment of Zhejiang Province School of Mechanical Engineering Zhejiang University Hangzhou 310027 China; ^5^ Qiushi Academy for Advanced Studies Zhejiang University Hangzhou 310027 China; ^6^ Eye Center the Second Affiliated Hospital School of Medicine Zhejiang University Hangzhou 310009 China; ^7^ Zhejiang SCI‐TECH University Hangzhou 310018 China; ^8^ School of Pharmaceutical Sciences Hangzhou Medical College Hangzhou 310013 China; ^9^ Institute for Regenerative Medicine Shanghai East Hospital School of Life Sciences and Technology Tongji University Shanghai 200123 China; ^10^ Shanghai Engineering Research Center of Stem Cells Translational Medicine Shanghai 200335 China

**Keywords:** 3D printing, cell delivery, human amniotic epithelial stem cells, hydrogel scaffolds, spinal cord injury

## Abstract

Stem cells play critical roles in cell therapies and tissue engineering for nerve repair. However, achieving effective delivery of high cell density remains a challenge. Here, a novel cell delivery platform termed the hyper expansion scaffold (HES) is developed to enable high cell loading. HES facilitated self‐promoted and efficient cell absorption via a dual driving force model. In vitro tests revealed that the HES rapidly expanded 80‐fold in size upon absorbing 2.6 million human amniotic epithelial stem cells (hAESCs) within 2 min, representing over a 400% increase in loading capacity versus controls. This enhanced uptake benefited from macroscopic swelling forces as well as microscale capillary action. In spinal cord injury (SCI) rats, HES–hAESCs promoted functional recovery and axonal projection by reducing neuroinflammation and improving the neurotrophic microenvironment surrounding the lesions. In summary, the dual driving forces model provides a new rationale for engineering hydrogel scaffolds to facilitate self‐promoted cell absorption. The HES platform demonstrates great potential as a powerful and efficient vehicle for delivering high densities of hAESCs to promote clinical treatment and repair of SCI.

## Graphical abstract

In this study, a high cell‐loaded delivery system with self‐promoted cell absorption to address current drawbacks in cell‐involved tissue engineering therapeutics and to match future clinical translation is developed. Standard hAESCs agent, consumable‐like HES and user‐friendly operation will jointly fuel tremendous progress in future SCI repair, which will also be promising for applications and therapies in various diseases.

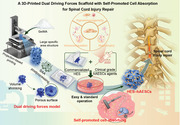

## 1 Introduction

**Figure 1:**
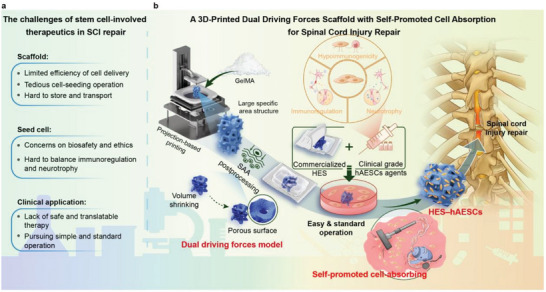
Strategy proposal of dual driving forces model and HES–hAESCs for SCI repair. a) Current challenges faced by stem cell‐involved therapeutics are listed from the perspectives of scaffold, seed cell and practical requirement in clinic. b) Sketch of the proposed strategy. Briefly, GelMA scaffolds with large specific surface area were manufactured to increase the upper limit of cell loading. Then, SAA postprocessing conferred HES with dual driving forces for self‐promoted cell‐absorbing in rehydration. After that, HES are easy for long‐term storage and transportation. HES rapidly absorbs 2.6 million cells in ≈2 min. HES–hAESCs is then used for SCI repair by the immunoregulation and neurotrophy of hAESCs. HES functions in a similar way to a vacuum cleaner by efficiently absorbing hAESCs driven by dual driving forces. Scaffolds volume: a 50–60 mm^3^ GelMA scaffold is designed and printed; After SAA postprocessing, the scaffold shrinks to ≈1 mm^3^ and can be packaged independently; After rehydration in cell suspension, the volume of HES–hAESCs returns back to ≈50 mm^3^. Abbreviations: SCI, spinal cord injury; TPMS, three‐period minimal surface; GelMA, gelatin‐methacryloyl; SAA, super absorption and attachment; HES, hyper expansion scaffold; hAESCs, human amniotic epithelial stem cells; HES–hAESCs, high hAESCs‐loaded HES.

Spinal cord injury (SCI) is a serious disease of the central nervous system with more than half a million new cases worldwide reported every year.^[^ ^1^, ^2^ ^]^ The main contributors to poor recovery from SCI are a nonpermissive microenvironment and insufficient replenishment of cells in the lesion.^[^ ^3^, ^4^ ^]^ To date, no safe and effective clinical therapy could strike a balance between inhibiting neuroinflammation and restoring neural circuits after SCI.^[^ ^5^, ^6^, ^7^ ^]^ Over the last decade, significant progress has been made toward elucidating the molecular and cellular mechanisms governing SCI pathology, as well as understanding the limitations of reparative strategies relying solely on biomaterials or stem cell transplantation.^[^ ^8^, ^9^, ^10^, ^11^, ^12^ ^]^ Hence, biomedical engineering shows potential for developing effective SCI treatments.^[^ ^13^ ^]^ Nevertheless, addressing persistent challenges related to scaffolds design, seed cells, and translational applications remains an important goal (**Figure** **1**a).

There is an urgent need to develop improved scaffold‐based cell delivery systems to solve the issues regarding insufficient cell loading and operational complexity within current approaches.^[^ ^14^, ^15^ ^]^ Due to the high flow resistance in the depths of scaffolds, surface utilization is poor with a nonhomogeneous distribution of cells. Cell‐laden bioprinting enables rapid production of customized implants containing highly dense and precisely arranged cells.^[^ ^16^, ^17^, ^18^, ^19^ ^]^ But cells enclosed within bioinks face risks of compromised viability, activity and DNA stability from unavoidable mechanical stress and free radical during printing and molding, particularly for delicate cell types like stem cells and neuronal cells.^[^ ^20^, ^21^, ^22^, ^23^ ^]^ Overcoming this challenge is critical to enable translational applications. Many cell‐friendly processes and parameters were established to minimize the cellular damage during fabrication,^[^ ^24^, ^25^ ^]^ however, it may require more improvements in techniques for practical use. Thus, separating cells from scaffold fabrication could be a proper strategy, improving the modularization and standardization for practical applications.^[^ ^26^ ^]^

While induced pluripotent stem cells (iPSCs) and their derived cells remain popular candidates in many preclinical studies dur to their versatility,^[^ ^27^ ^]^ concerns regarding safety and ethics impede their clinical translation.^[^ ^28^, ^29^ ^]^ Given the neural cell loss and severe neuroinflammation after SCI, identifying candidate cells that can balance immunomodulation and neural properties is critical for repair. Our previous studies have demonstrated that human amniotic epithelial stem cells (hAESCs), which are perinatal stem cells from the amnion of the placenta, possess critical properties of guaranteed safety, hypoimmunogenicity and immunoregulation.^[^ ^30^, ^31^, ^32^, ^33^, ^34^, ^35^ ^]^ Additionally, hAESCs secrete several anti‐inflammatory and neurotrophic factors, highlighting their potential for use in SCI treatment.^[^ ^36^, ^37^, ^38^, ^39^ ^]^

To address these issues, we proposed a dual driving forces model to explain the mechanism of efficient cell absorption. Based on this model, we developed a delivery tool with high cell loading via self‐promoted absorption, which we termed hyper expansion scaffold (HES). Considering the suitable properties of hAESCs for SCI treatment, we further established a novel therapeutic system named high‐hAESCs‐loaded HES (HES–hAESCs), and assessed its effects in SCI models (Figure 1b). Gelatin‐methacryloyl (GelMA) scaffolds with high specific surface area were dehydrated through a super‐absorption and adhesion (SAA) postprocessing. The dehydrated scaffolds facilitated highly efficient cell absorption via two synergistic driving forces: macroscopic swelling and microscopic features. Through this mechanism, HES achieved loading of 2.6 million uniformly distributed cells (≈5.2 × 10^4^ cells mm^−3^) within a 2 min immersion in hAESCs suspension, rendering it a delivery tool with high hAESCs loading for SCI repair. In rat SCI models, HES–hAESCs treatment significantly improved hindlimb locomotion, as shown by discontinuous weight‐bearing crawling and active signals of motor evoked potential (MEP). The treatment also contributed to axonal projections and preservation of myelination. The underlying mechanisms involved hAESCs‐mediated inhibition of the immune response, and increased levels of neurotrophic factors around the lesions. These findings indicate that HES–hAESCs effectively improved the tissue microenvironment and ameliorated motor deficits in SCI rats.

The proposed dual driving forces model provides a novel approach and underlying mechanism to enable self‐promoted cell uptake by hydrogel scaffolds. HES can serve as an efficient delivery tool to load multiple cell types. Additionally, it can also be packaged independently and manufactured into products suitable for long‐term storage and convenient clinical administration. Furthermore, hAESCs may be developed into a cellular agent compliant with good manufacturing practice, based on safety evaluation testing and clinical‐graded preparation. Taken together, our work provides a clinically‐translatable approach with potential for treating SCI.

## 2 Results

### 2.1 Precise Manufacturing of Scaffolds with a Large Specific Surface Area

**Figure 2:**
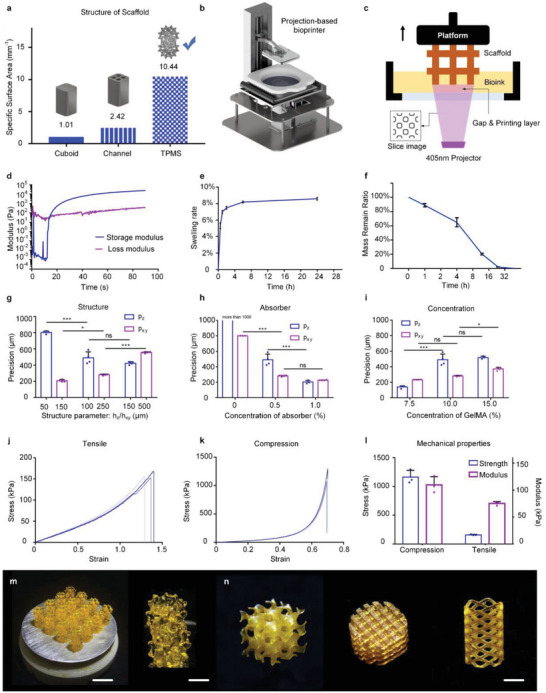
Design and 3D printing of scaffolds with large specific surface area. a) TPMS structure has the largest specific surface area compared to other structures. b) Projection‐based bioprinter. c) Manufacturing principle of projection‐based bioprinting. d) Changes of storage modulus and loss modulus of GelMA hydrogel during photo crosslinking. e) Swelling rate of GelMA hydrogel in PBS. *n* = 4; error bars, SEM. f) Degradation of GelMA hydrogel by type II collagenase in vitro. *n* = 3; error bars, SEM. g) The relationship between structure parameter and printing precision in x‐y plane and along z direction. h) The relationship between concentration of light absorber and printing precision in x‐y plane and along z direction. i) The relationship between concentration of GelMA solution and printing precision in x‐y plane and along z direction. g–i) One‐way ANOVA, followed by Tukey post‐hoc test. *n* = 4; ***p < 0.001; **p < 0.01; *p < 0.05; error bars, SEM. j) Tensile curve of GelMA hydrogel. k) Compression curve of GelMA hydrogel. l) Tensile/compression modulus/strength of GelMA hydrogel. *n* = 4; error bars, SEM. m) Simultaneous printing of scaffolds with large specific surface area. Scale bars: 1 cm, 2 mm. n) Other scaffolds with TPMS structure printed with fine precision. Scale bars: 1 cm. Abbreviations: TPMS, three‐period minimal surface.

To achieve high and efficient cell loading within a unit volume scaffold, our focus was twofold: increasing the specific surface area and improving surface utilization in deep scaffolds. Initially, we designed a three‐period minimal surface (TPMS) characterized by interconnected pores, continuous and smooth 3D surfaces, and a large specific surface area. These structural advantages render the TPMS highly suitable for cell adhesion and circuit connections during SCI repair. When compared to the conventional poured solid structure and the general structure with channels, TPMS exhibits surface areas that are five and ten times larger specific surface area, respectively (**Figure** **2**a).

Projection‐based 3D bioprinting (PBP) offers high precision, fast formation, and good repeatability, making it suitable for the fabrication of TPMS scaffolds (Figure 2b,c). GelMA is a photocurable and biocompatible hydrogel that can cross‐link under specific light conditions, and thus an ideal material for PBP. To ensure accurate fabrication, we determined the printability of GelMA‐PBP, and conducted a rheological property test to characterize state changes during crosslinking. Under intense printing light, GelMA reached the gel point (the intersection of G′ and G″ curves, Figure 2d) in ≈20 s, making it suitable for projection‐based bioprinting. The swelling property test indicated a maximum volume change rate of ≈8.5% upon reaching osmotic equilibrium within 6 h (Figure 2e). Hence, no significant errors were incurred. Moreover, GelMA was completely degraded by 50 U mL^−1^ type II collagenase after 32 h (Figure 2f), demonstrating its good biodegradability.

To evaluate the impact of process parameters on errors, we utilized standard assessment methods.^[^ ^40^, ^41^ ^]^ The radial shape model was used to analyze the error within the x‐y plane, and the spiral staircase shape model was used for the z‐direction. Each model accurately calculated the current error values based on the model design parameters and only one measurement value. Using this approach, we examined the effect of three important parameters on printing errors: structural parameters, light absorber concentration, and GelMA material concentration (Figure 2g–i). Structural parameters could guide wall thickness design in TPMS scaffolds. Based on Figure 2g, we designed a structure with a wall thickness of 300 µm. The addition of a light absorber reduced light scattering and transmission, and thus significantly improved the printing accuracy. As Figure 2h showed, a 1% (w/v) light absorber concentration was selected. Figure 2i indicated GelMA concentration above 10% had a minimal impact on printing accuracy. Therefore, 15% was selected to meet the requirements of mechanical properties.

The mechanical properties were evaluated using a previously reported method.^[^ ^40^ ^]^ The tensile and compressive strains reached 125% (Figure 2j) and 68% (Figure 2k), respectively. These results indicated that the scaffold could withstand the deformation caused by subsequent surgeries. A significant difference was observed in the modulus at 10% strain and destructive strength between the compression and tensile tests (Figure 2l). In the SCI model, the scaffold was not sutured to lesions. Therefore, the TPMS scaffold was mainly subjected to compression rather than tension from surrounding tissues.

Based on these results, we manufactured TPMS scaffolds with large specific surface area to increase cell‐loading capacity (Figure 2m,n).

### 2.2 Development of Dual Driving Forces Model and SAA Postprocessing

**Figure 3:**
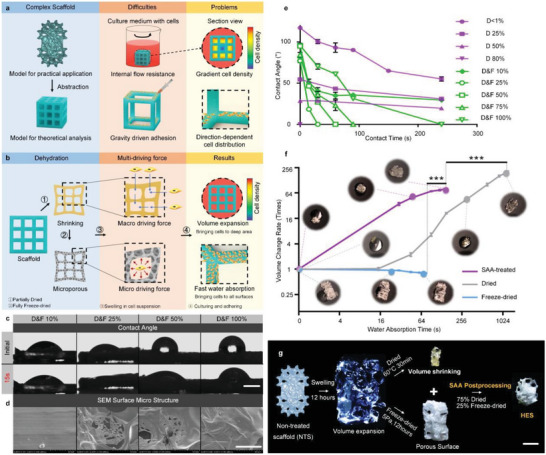
The dual driving forces model, SAA postprocessing, and the rapid water absorption of HES. a) Drawbacks of traditional cell‐seeding method applied in 3D complex structures. b) The dehydration strategy for providing macroscopic and microscopic driving force for self‐promoted cell absorption. c) Contact angle of all samples treated with different drying/freeze‐drying rate. Scale bar: 500 µm. d) SEM images of samples with different drying/freeze‐drying rate. Scale bar: 50 µm. e) Contact angle changing over time during water absorbing; *n* = 4; error bars, SEM. f) The water absorbing rate and swelling volume of TPMS scaffolds with different postprocessing. One‐way ANOVA, followed by Tukey post‐hoc test. *n* = 3; ***p < 0.001; error bars, SEM. g) SAA postprocessing illustration and images contrast among NTS, dried scaffold, freeze‐dried scaffold and HES. Scale bar: 1 mm. Abbreviations: D&F: dried and freeze‐dried; SEM: scanning electron microscope; NTS: non‐treated scaffold; SAA, super absorption and attachment; HES, hyper expansion scaffold.

Although precisely fabricated TPMS GelMA hydrogel scaffolds exhibited a significantly increased cell‐carrying capacity, these hydrogels still lack satisfactory surface utilization and cell distribution levels, especially due to high flow resistance and gravity (**Figure** **3**a). In 3D space, cells tend to attach to surfaces perpendicular to gravity, and thereby result in an uneven distribution of cells on other surfaces, which reduces surface utilization within the scaffold. (Figure S1a,b, Supporting Information). These issues cannot be avoided even with different cell seeding methods, such as injection or dropwise addition of cell suspensions (Figure S1c,d, Supporting Information).

To address this issue, we dehydrated hydrogel scaffolds, and proposed a dual driving forces model for efficient cell absorption. The scaffolds contracted after drying in an air oven. Volume swelling during rehydration is the macroscopic driving force of absorption. On the other hand, vacuum freeze‐drying led to a porous surface of the hydrogel scaffolds, providing a microscopic driving force via capillarity. These two driving forces ultimately resulted in a homogeneous distribution of cells on the surface of the scaffolds (Figure 3b).

To test this hypothesis, we first tested the surface hydrophilicity of hydrogel sheets with different dehydration treatments using contact angle test. Hydrophilia declined rapidly with decreasing water content in the hydrogels treated only by drying. The completely dried surface exhibited super hydrophobicity, which was detrimental to cell attachment (Figure S2, Supporting Information). We applied different treatment strategies by combining drying and freeze‐drying. For instance, 90% of the water was removed by drying, and the remaining 10% was removed by freeze‐drying (named D&F 10%). Figure 3c demonstrated that surface of D&F 25% exhibited the smallest initial contact angle among all samples. After 15 s, the droplet on the D&F 25% surface was completely absorbed compared with the large contact angles on the other sheets. The scanning electron microscope (SEM) images further explained the phenomena from the perspective of the surface microstructure. SEM results demonstrated that, in addition to the countless microchambers formed by the sublimated ice crystals during freeze‐drying, many cavities were also found on the walls between the chambers throughout the whole sample (Figure 3d; Figure S3, Supporting Information). Additionally, the number of cavities was found to be related to the freeze‐drying and drying rate. These cavities connect the microchambers to each other, allowing water to flow quickly into deep areas. Therefore, water filled the chambers in a short time, and the large surface area of the water‐free hydrogel began to seep. When rehydration occurred, the absorption speed increased rapidly with decreasing water content. This phenomenon is conducive to water absorption, and forms the positive feedback to accelerate this process.

Subsequently, a line chart was constructed to more intuitively depict changes in contact angle over time. The D&F 25% surface was found to possess distinct hydrophilicity and water‐absorption, as evidenced by the smallest initial contact angle and fastest absorption rate among all fully dehydrated samples (Figure 3e). hAESCs were seeded onto hydrogel sheets from different treatment groups to test whether hydrophilicity and water‐absorbing capacity promote cell loading and attachment. The results showed the group treated with D&F 25% had a significant advantage over both the non‐treated scaffold (NTS) and D<1% groups, and this advantage in cell loading and attachment increased over time (Figure S4a, Supporting Information). Moreover, quantitative analysis of cell density on different hydrogel surfaces also supported the fluorescence results, demonstrating that an initial cell loading density of 663.7 cells mm^−2^ with D&F 25%, which reached 3169 cells mm^−2^ on day 7 (Figure S4b, Supporting Information). We thus termed this “super‐absorption and adhesion” (SAA) postprocessing. Specifically, a total of 75% of water was removed from NTS by drying. The loss of water caused significant volume shrinkage, which is more than 100 times that of the TPMS structure. The shrunken scaffolds were freeze‐dried to remove the remaining 25% water. Here, the remaining water gradually condenses into ice crystals uniformly distributed inside and on the surface of the scaffold. During vacuuming, these ice crystals sublimate, leaving many cavities in situ. Numerous microscopic pores further increase the specific surface area and hydrophilicity.

To compare the absorption performances, 100 µL of PBS buffer was dropped onto the dried, freeze‐dried, and SAA‐treated TPMS scaffolds (Figure 3f; Figure S5, Supporting Information). The volume of the SAA‐treated scaffold dilated over 80 times in 150 s. The dried scaffold required nearly 20 min to complete absorption owing to its highly hydrophobic surface, and the freeze‐dried scaffold yielded a negative volume change rate, which had no effect on the formation of a macroscopic driving force. Consequently, SAA postprocessing enabled the hydrogel scaffold to obtain a hydrophilic surface, significant volume change, and porous microstructure to efficiently absorb liquids based on the dual driving forces mechanism. We termed this SAA‐treated TPMS scaffold as hyper expansion scaffold (HES).

Taken together, we proposed a dual driving forces model, and elucidated its mechanism for efficient water absorption. Based on these results, we optimized the dehydration methods and developed SAA postprocessing to manufacture HES by combining the advantages of drying and freeze‐drying to achieve rapid water absorption by scaffold (Figure 3g). Next, we tested the scaffold by cell experiments in vitro.

### 2.3 Establishment of Efficient and Uniformly Distributed HES–hAESCs Delivery System

**Figure 4:**
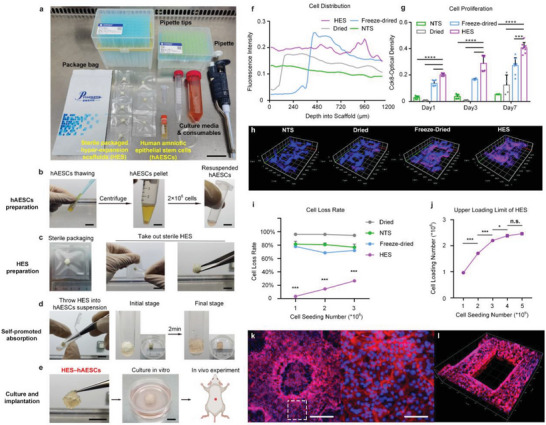
HES–hAESCs delivery system with high cell‐loading and uniform distribution. a) All items used in the preparation of HES–hAESCs. Scale bar: 4 cm. b–e) The procedure included four steps. b) hAESCs preparation included cell thawing, centrifugation and resuspending hAESCs to make ≈200 µL suspension. Scale bar: 2 cm, 700 µm, 700 µm. c) Sterile HES with independent package. Scale bar: 2 cm, 500 µm. d) Self‐promoted absorbing capacity of HES improved efficiency of cell seeding. About 2 min after throwing HES into suspension, cell seeding is completed. Scale bar: 1 cm. e) Culture the HES–hAESCs for following applications (optional). Scale bar: 1 cm, 700 µm. f) Fluorescence intensity of cells throughout the scaffolds 1 day after seeding. g) hAESCs proliferation (CCK8) on different scaffolds. One‐way ANOVA, followed by Tukey post‐hoc test. *n* = 8; ****p<0.0001; ***p < 0.001; **p < 0.01; *p < 0.05; error bars, SEM. h) The fluorescence images of GelMA (blue) and hAESCs (red) on different scaffolds. HES carried more cells and exhibited uniform distribution. i) Cell loss rate test to assess the hAESCs‐loading efficiency on different scaffolds in a 2‐min process of cell seeding. One‐way ANOVA, followed by Tukey post‐hoc test. *n* = 5; ***p < 0.001; **p < 0.01; *p < 0.05; error bars, SEM (error bars in dried, freeze‐dried and HES groups didn't show, due to the large spaced ticks of Y axis). j) Upper limit of cell loading number on proper size of HES for rat SCI repair. One‐way ANOVA, followed by Tukey post‐hoc test. *n* = 5; ***p < 0.001; **p < 0.01; *p < 0.05; error bars, SEM. k) The fluorescence images of hAESCs (DAPI/F‐actin) on HES with TPMS structure. Scale bar: 250 µm, 50 µm. l) Dense hAESCs (DAPI/F‐actin) attached uniformly on SAA‐treated scaffold with regular stereoscopic structure.

The need to improve the efficiency of the tedious cell seeding and cell‐loading on scaffolds has been recognized.^[^ ^3^, ^15^ ^]^ To determine whether HES improves the efficiency of cell seeding, we recorded the entire process of seeding of hAESCs onto the HES. **Figure** **4**a vividly showed all items used in this process, including sterile packaged HES and hAESCs. The procedure was divided into four parts: hAESCs and HES preparation, self‐promoted absorption, cultivation and implantation (Figure 4b–e). For practical use, hAESCs were thawed, and the proper number of cells was resuspended in a tube (according to the treatment requirement and size of the HES). Full dehydration facilitated storage and transport of sterile and independently packaged HES. The HES was then removed and immersed in the hAESCs suspension. After 2 min, the HES was expanded and filled with hAESCs, which was termed HES–hAESCs. Using this procedure, HES–hAESCs can be implanted immediately during an emergency, or cultured for several days until the most appropriate time for intervention.

We then evaluated the cell viability, loading, and distribution of HES–hAESCs. Flow cytometric analysis indicated a much better cell viability on HES–hAESCs than that on cell‐laden printing (Figure S6a,b, Supporting Information). Moreover, rhodamine‐marked hAESCs were uniformly distributed throughout the HES from the surface to depths, as demonstrated by quantitative fluorescence statistics along the depth direction (Figure 4f). The CCK8 test indicated that hAESCs seeded on the HES exhibited the most robust proliferation and cell loading among all treated scaffolds (Figure 4g), which was consistent with the results of fluorescence staining (Figure 4h). Furthermore, hAESCs loading efficiency was assessed using the loss rate test, which demonstrated that only 3.42% of 1 million hAESCs were left unabsorbed by the HES. In contrast, 96.14% and 81.40% of the hAESCs were unabsorbed by the dried scaffold and NTS, respectively. The cell loss rate on the HES was maintained at a relative minimum even when 3 million hAESCs were seeded (Figure 4i). Notably, the loading limit of the HES was close to 2.6 million hAESCs, which was increased by 2 million cells compared with NTS (Figure 4j). Therefore, the cell density reached more than 5 × 10^4^ cells mm^−3^ in the HES with the proper volume for rat SCI repair. Immunofluorescence staining further indicated that hAESCs fully covered the curved surface of the HES, and exhibited robust growth (Figure 4k) even on SAA‐treated scaffolds with regular stereoscopic structures (Figure 4l). We printed HES scaffolds of different sizes to assess their cell loading capacity (Figure S7a, Supporting Information). Size C scaffold, with a volume of ≈200 mm^3^, was found to be able to load more than 8.7 million hAESCs (Figure S7b, Supporting Information). Considering the actual size of the human spinal cord, the volumes of the SCI lesion and implanted scaffold in patients were much larger than those in rats. Thus, a scaffold suitable for human SCI wounds should carry tens of millions of cells with a cell density of ≈5 × 10^7^ cells cm^−3^.

Overall, these findings demonstrated that the HES delivery system was able to load high‐density and uniformly distributed hAESCs loading via an efficient self‐promoted absorption for in vivo applications. Additionally, the user‐friendly procedure contributes to the standardization of clinical use combined with commercialized HES and clinical‐grade hAESCs agent.

### 2.4 Implantation Feasibility of HES–hAESCs in Rat SCI Models

**Figure 5:**
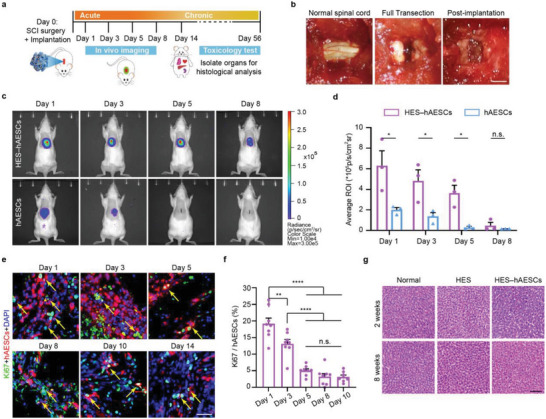
Implantation feasibility of HES–hAESCs in rat SCI model. a) Experimental schematic. After T10 surgery and implantation, rats were implemented luciferase bio‐imaging within 8 days, and histological evaluation of vital organs at 2 and 8 weeks. b) Representative images of implantation surgery. c) Representative whole‐body bioluminescence images of rats after transplanting luciferase over‐expressed hAESCs. d) Quantitative analysis of bioluminescence in rats at different time points. Student’ s t‐test. *n* = 3 rats; *p < 0.05; error bars, SEM. e) Ki67 staining revealed that a certain percentage of hAESCs maintained proliferation 2 weeks post‐implantation. Yellow arrows showed hAESCs (red) labeled with Ki67 (green). Scale bar: 20 µm. f) Quantitative analysis of the percentage of hAESCs labeled with Ki67. One‐way ANOVA, followed by Tukey's post‐tests. *n* = 8 sections from 3 rats per group; ****p < 0.0001; **p < 0.001; error bars, SEM. g) HE staining of livers at 2 weeks (acute) and 8 weeks (chronic) post‐implantation. Scale bar: 100 µm.

Prior to evaluating therapeutic effects, we assessed the implantation feasibility of HES–hAESCs in rats by conducting in vivo cell survival and toxicology tests (**Figure** **5**a). While many studies implemented graft 2 weeks post‐injury due to the toxic and inflammatory environment at acute lesion sites, scaffolds could protect and support grafted cells survival in acute SCI.^[^ ^3^, ^13^ ^]^

To test the feasibility of our approach, we performed T10 laminectomy on Sprague‐Dawley rats and replaced a 2–3 mm block of the spinal cord with a dissociated hAESCs suspension and HES–hAESCs in the acute SCI phase (Figure 5b). Luciferase imaging demonstrated that a strong biosignal was detected in rats implanted with HES–hAESCs for more than 1 week. In contrast, the signal in the hAESCs‐treated rats dwindled rapidly within 3 days (Figure 5c). Statistical analysis indicated that the signal intensity in HES–hAESCs rats was significantly higher than that in the hAESCs‐treated group within 1 week, indicating significant protection by the scaffold for cell survival in the lesions (Figure 5d). Moreover, the immunofluorescence of Ki67 in prelabeled hAESCs suggested that a certain number of hAESCs could maintain their proliferation for 2 weeks (Figure 5e). Given the harsh microenvironment for hAESCs proliferation, the real ratio of cell survival was much higher than the staining ratio by Ki67 (Figure 5f). To evaluate the safety of our treatment approach, histological analysis of certain vital organs was conducted at 2 and 8 weeks post‐implantation. Hematoxylin and eosin staining of the organs revealed normal histology at both time points, indicating that grafts were well‐tolerated across species (Figure 5g; Figure S8a,b, Supporting Information).

These data demonstrated the long‐term safety and stability of the graft system after hetero‐transplantation, increasing its potential therapeutic effect.

### 2.5 HES–hAESCs Restore Hindlimb Locomotion in SCI Rats

**Figure 6:**
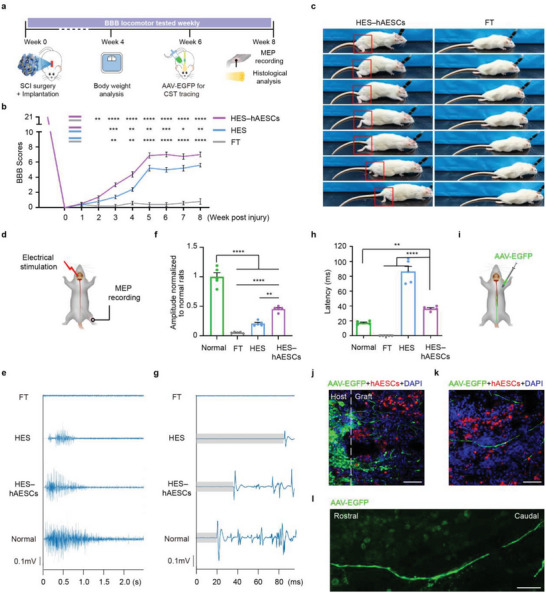
Eight‐week period motor function from in vivo studies of HES–hAESCs. a) Experimental schematic. After T10 surgery and implantation, rats underwent the body weight measurement at 4 weeks and 8 weeks post‐surgery. AAV‐EGFP was injected into upper spinal cord (T7) to trace the corticospinal tract (CST) at 6 weeks, and MEP and histological test were implemented at 8 weeks post‐surgery. b) BBB locomotion scores within 8 weeks. One‐way ANOVA, followed by Tukey post‐hoc test. *n* = 5 rats; ****p < 0.0001; ***p < 0.001; **p < 0.01; *p < 0.05; error bars, SEM. c) Representative images of hindlimbs locomotion while crawling freely, red frames show the whole stepping gait of HES–hAESCs rats. d) Schematic diagram of the MEP study performed at 8 weeks post‐surgery. Transcranial electrical stimulation is implemented to the motor cortex in the brain and MEPs are recorded from the contralateral hindlimbs. e) Representative responses in the TA muscle evoked by epidural motor cortex stimulation from indicated groups. f) Quantitative analysis of the MEP response amplitude from indicated groups. One‐way ANOVA, followed by Tukey post‐hoc test. *n* = 5 rats; ****p < 0.0001; error bars, SEM. g) Representative images to show the MEP responses latency from indicated groups. Gray part showed the latency period. h) Quantitative analysis of the response latency from indicated groups. One‐way ANOVA, followed by Tukey post‐hoc test. *n* = 5 rats; ****p < 0.0001; **p < 0.01; error bars, SEM. i) Schematic diagram of injection AAV‐EGFP into T7 spinal cord performed at 6 weeks post‐surgery. j) EGFP signal was present in the rostral interface between host tissue and HES–hAESCs grafts, and some of that entered the scaffolds and descended through the lesion center. hAESCs were prelabeled with red fluorescence before implantation. Scale bar: 150 µm. k) Descending EGFP axons were detected in the middle of lesion, which was more than 1 mm away from the rostral interface. Scale bar: 50 µm. l) Magnified image showed the length of descending EGFP axon for more than 400 µm. Scale bar: 50 µm.

To determine the effects of HES–hAESCs on general health and hindlimb locomotion recovery in vivo, SCI rats underwent assessment of body weight, Basso, Beattie and Bresnahan (BBB) locomotor scale scoring, motor evoked potential (MEP), and corticospinal tracing (CST) over an 8‐week period (**Figure** **6**a). The treatment groups were as follows: normal rats (normal), full transection SCI rats (FT), SCI rats treated with empty HES (HES), and high hAESCs‐loaded HES (HES–hAESCs).

Postoperative weight statistics indicated that rats treated with HES–hAESCs were healthier than FT rats at weeks 4 and 8 (Figure S9a,b, Supporting Information). Additionally, HES–hAESCs rats exhibited significantly higher locomotor recovery compared to those implanted with empty HES at 3 weeks after transplantation (Figure 6b), according to functional scoring described previously.^[^ ^13^, ^42^ ^]^ Specifically, HES–hAESCs rats reached a mean value of 7.0 ± 0.3 points (± s.e.m.) at 8 weeks post‐procedure, indicating extensive movement in approximately three joints of the hindlimb. In contrast, HES rats demonstrated only slight movement of two or three joints, and no obvious movement was observed in the FT controls, with a mean score of 0.8 ± 0.3 (± s.e.m.). Moreover, the implantation of hAESCs (cells suspension) showed only negligible improvement in locomotion recovery compared to FT rats (Figure S9c, Supporting Information), suggesting that hAESCs without scaffold support have limited functionality in the gap lesions of SCI. Therefore, the hAESCs‐treated rats were excluded from further experiments. We also recorded the gait and electromyograph (EMG) of the hindlimbs of the rats while crawling freely in an open field at 8 weeks post‐implantation. HES–hAESCs rats exhibited continuous steps of weight‐bearing walking on the hind palms (Figure 6c and Video S3, Supporting Information) compared with the dragging of hindlimbs in the FT group (Video S4, Supporting Information). EMG data showed improved amplitude and frequency in HES–hAESCs rats, suggesting an improvement in hindlimbs locomotion, which is consistent with the BBB scores as well (Figure S9d, Supporting Information).

We further investigated the MEP signals of the hindlimbs to quantitatively assess the functional recovery and the degree of neural circuits connections (Figure 6d). EMG activity in the tibialis anterior (ankle flexor) muscle was detected in HES–hAESCs and HES rats, whereas no signal was observed in FT rats after stimulation. Thus, no functional neural circuit reconstruction could be achieved spontaneously in the SCI rats (Figure 6e). Importantly, the EMG activity in HES–hAESCs rats was significantly greater than that in HES rats, with the former reaching 40%–45% of the EMG activity of normal rats. This result further demonstrates that HES–hAESCs have a positive effect on the reconnection of descending neural circuits (Figure 6f). Moreover, the latency of MEP signals in HES–hAESCs rats was shortened after stimulation, allowing the rats to act agilely (Figure 6g,h).

To examine the propriospinal axons descending through the lesion center, AAV2/9‐EGFP was injected into the upper thoracic spinal cord (T7) of rats two weeks before sacrifice (Figure 6i), as described elsewhere.^[^ ^43^ ^]^ Few EGFP‐labeled axons were observed in the lesion areas of the HES or FT rats (Figure S9e, Supporting Information). However, in HES–hAESCs rats, EGFP signals were abundant at the rostral interface between the host and the graft, and some of these signals were caudally projected into the inner channels of the scaffolds (Figure 6j), with a maximum distance of more than 1.5 mm and a continuous length of ≈400 µm (Figure 6k,l). Additionally, we found a corresponding positional relationship between hAESCs and descending nerve fibers in the scaffold channels, suggesting that nerve fibers may interact with hAESCs for neurotrophy or nourishment to promote projection and extension (Figure S9f, Supporting Information). These findings demonstrates that the repair of descending neural circuits could be improved by HES–hAESCs, which mediate the recovery of hindlimbs locomotion in rats.

Urinary system function is also critical in SCI repair. Analysis of bladder weight (Figure S10a,b, Supporting Information) and histology (Figure S10c, Supporting Information) revealed that HES–hAESCs significantly inhibited the pathological enlargement of the bladder, although differences from the normal bladder were also noted. This indicates that the intervention mitigated ‐to some extent‐ the structural damage to the bladder caused by urinary retention.

### 2.6 HES–hAESCs Promote the Development and Maturation of Nerve Fibers in SCI Lesion

Morphological analysis revealed that FT controls developed an atrophic lesion structure consistent with chronic injury pathology. In contrast, no atrophy occurred in two treatment groups, with the grafts tightly attached to the host tissues both rostrally and caudally (Figure S11a,b, Supporting Information). HE staining exhibited that lesions of HES–hAESCs were relatively robust and dense, unlike cavitary lesions in FT rats. We also found that the remaining scaffolds were stained dark purple, suggesting that the implanted scaffold functioned as a physical support for more than 8 weeks (Figure S11c, Supporting Information).

**Figure 7:**
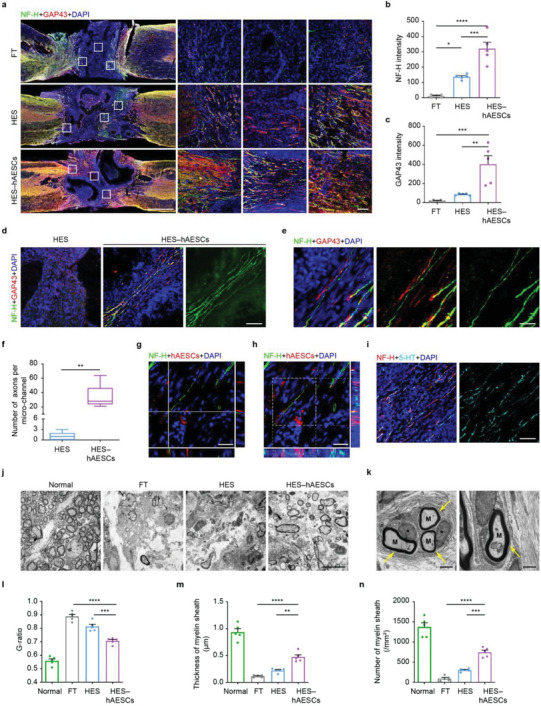
Improved growth and myelination of nerve fibers triggered by HES–hAESCs. a) The fluorescence images of NF‐H (green) and GAP43 (red)‐labeled axons showed the growth of nerve fibers around the lesion from indicated groups. Right side shows the magnified areas from white frames in images on the left. Scale bars: 1 mm, 50 µm. b,c) Quantitative analysis of the fluorescence intensity of NF‐H and GAP43 from indicated groups respectively. One‐way ANOVA, followed by Tukey post‐hoc test. *n* = 5; ****p < 0.0001; ***p < 0.001; **p < 0.005; *p < 0.05; error bars, SEM. d) The growth and projection of nerve fibers in microchannels between empty HES and HES–hAESCs rats at 8 weeks post‐implant. Scale bar: 50 µm. e) Magnified images of nerve fibers labeled with NF‐H and GAP43 in HES–hAESCs rats. Scale bar: 15 µm. f) Quantitative analysis of the mean number of axons per microchannel. Student’ s t‐test. *n* = 5; **p < 0.005; error bars, SEM. g,h) The fluorescence images indicated that NF‐H‐labeled axons projected through the channels filled with hAESCs. Scale bars: 30 µm. i) The fluorescence images indicated that certain mature axons (NF‐H positive) also expressed 5‐HT, suggesting the presence of specific subtype of neurons. Scale bars: 30 µm. j) The ultrastructural images showed the myelin sheath structure in the lesions from the indicated groups. Scale bars: 30 µm. k) Magnified views of myelin sheath and surrounding oligodendrocytes in the lesion of rats implemented with HES–hAESCs. M: myelin sheath (yellow arrows), *oligodendrocytes. Scale bars: 2 µm, 1 µm. l–n) Statistical analysis of G‐ratio, thickness and number of myelin sheath in the lesions from indicated groups. One‐way ANOVA, followed by Tukey post‐hoc test. *n* = 5; ****p < 0.0001; ***p < 0.001; **p < 0.01; error bars, SEM.

Large numbers of NF‐H and GAP43‐labeled axons were aligned into linear arrays in the HES channels loaded with hAESCs, adopting a pattern mimicking the linear neural alignment of native tissue (**Figure** **7**a). At the lesion epicenter, numerous developing nerve fibers filled with inner channels were observed in HES–hAESCs rats and some exhibited mature characteristics with NF‐H positivity. In contrast, few and disordered neural fibers were found in HES rats (Figure 7b,c). The robust and well‐organized pattern of axons in the lesion area contributed to the recovery of locomotion in HES–hAESCs rats.

We also found that the projection of nerve fibers in the microchannels of scaffolds in HES–hAESCs rats was significantly more vigorous than that in HES rats (Figure 7d). HES–hAESCs guided the projection of rostral and caudal axons and promoted their extension into depth. Magnified views demonstrated the robust morphology of nerve fibers in the internal channels (Figure 7e); the mean of 33 ± 7 axons per channel in HES–hAESCs rats were 10‐fold more than that in HES rats (Figure 7f). Moreover, NF‐H‐positive axons passed through the scaffold channels covered with hAESCs in view of the x–z and y–z sections, which is similar to the spatial position and function of cell nourishment in CST tracing (Figure 7g,h). Serotonergic axons, which modulate spinal motor systems, were also projected into the inner channels loaded with hAESCs and extended linearly from the rostral to caudal aspects of the scaffolds (Figure 7i).

Ultrastructural analysis of the axons in the hAESCs‐filled channels revealed a variety of myelin structures with multiple calibers (Figure 7j), and some with oligodendrocytes involved in myelination that formed the myelin sheath (Figure 7k, yellow arrows). Excitatory synapses with rounded synaptic vesicles were also observed in hAESCs‐loaded microchannels (Figure S12a, Supporting Information). Additionally, the R‐ratio and other parameters of myelin sheath statistics demonstrated that robust myelination occurred in HES–hAESCs rats, which was conducive to relaying and integrating nerve signals (Figure 7l–n; Figure S12b, Supporting Information). These findings are consistent with the results of Luxol fast blue staining (Figure S12c, Supporting Information). Therefore, HES–hAESCs could significantly preserve the degree of myelination in lesions after SCI and further contribute to the synaptic structures that relay descending neural outputs.

### 2.7 HES–hAESCs Inhibited Neuroinflammation and Improved the Neurotrophic Environment around SCI Lesion

**Figure 8:**
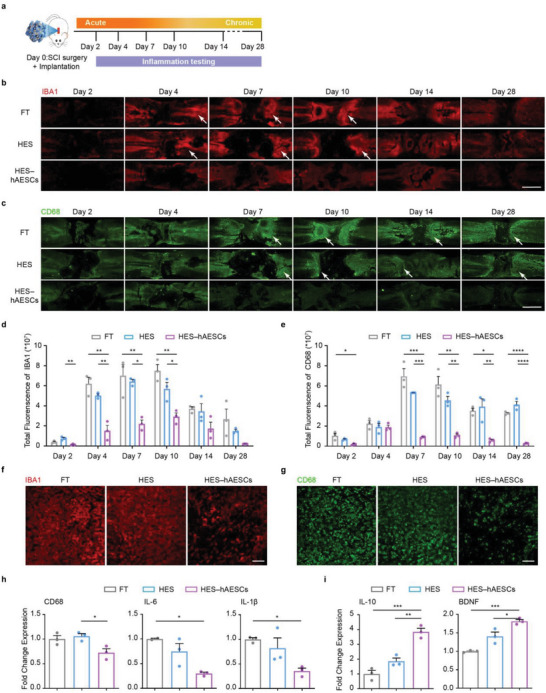
Inflammation regulation and neurotrophy effects on local tissues by HES–hAESCs. a) Experimental schematic. After T10 surgery and implantation, local inflammation and neurotrophy levels around lesions were examined from indicated groups ranging from acute injury phase to chronic phase. b,c) IBA1 and CD68 fluorescence staining showed the distribution and activity of microglia and microphage around lesions at different time points following implantation. White arrows show the presence and activity of microglia and macrophage around lesions in FT and HES groups. Scale bar: 2 mm. d,e) Quantitative analysis of fluorescence intensity of IBA1 and CD68 around the lesion epicenter at different time points from indicated groups. One‐way ANOVA, followed by Tukey post‐hoc test. *n* = 3; ****p < 0.0001; ***p < 0.001; **p < 0.01; *p < 0.05; error bars, SEM. f,g) Local views of IBA1 and CD68 around the interface between host spinal cord and grafts at 7 days post‐implant. Scale bars: 200 µm. h) Quantitative analysis of mRNA expression of inflammation‐related genes in the spinal cord tissues around lesions at 7 days post‐implant. One‐way ANOVA, followed by Tukey post‐hoc test. *n* = 3; *p < 0.05; error bars, SEM. i) Quantitative analysis of mRNA expression of anti‐inflammation and neurotrophy‐related genes in the spinal cord tissues around lesions at 7 days post implant. One‐way ANOVA, followed by Tukey post‐hoc test. *n* = 3; ***p < 0.001; **p < 0.01; *p < 0.05; error bars, SEM.

To explain mechanisms underlying the promotion of SCI repair by HES–hAESCs, we investigated the inflammatory responses and levels of neurotrophic factors around the injury epicenter over a 28‐day period based on the immunoregulation and paracrine properties of hAESCs (**Figure** **8**a). Neuroinflammation around lesions was evaluated over time at different time points, keeping all staining/imaging parameters consistent. HES–hAESCs significantly inhibited the activity of microglia and macrophages compared with HES and FT rats, as represented by the lower fluorescence of IBA1 and CD68 (Figure 8b,c). Statistical analysis demonstrated that HES–hAESCs inhibited the activation of microglia four days after transplantation (Figure 8d), whereas the inhibition of macrophages was mainly initiated approximately one week after injury (Figure 8e). Representative images of IBA1 and CD68 7 days post‐injury demonstrated significant effects of hAESCs on inflammation inhibition (Figure 8f,g). To our knowledge, the pro‐inflammatory property of macrophages peaks at one week post‐injury, and microglia are continuously reactivated after injury,^[^ ^43^ ^]^ indicating that hAESCs play a critical role in the tough phase.

Consistent with the change in CD68 expression, the expression of IL‐6 and IL‐1β was reduced in HES–hAESCs, as revealed by qPCR analysis (Figure 8h). Furthermore, increased levels of the anti‐inflammatory cytokine IL‐10 and neurotrophic factor BDNF demonstrated that an improved microenvironment for neural repair was created after implanting HES–hAESCs (Figure 8i). Therefore, we reasoned that hAESCs inhibit the activation of endogenous immune cells to suppress neuroinflammation after SCI. Increased neurotrophic levels further promote nerve growth for recovery of locomotion.

## 3 Discussion

The past decade has seen considerable advancement in SCI repair via 3D bioprinting, with various preclinical studies demonstrating effectiveness.^[^ ^13^, ^44^, ^45^, ^46^, ^47^ ^]^ However, translation of the most promising interventions in laboratory animals to clinical practice has been limited.

While various cell sources have been investigated, biosafety, ethics, and phenotypic matching are still the major concerns regarding clinical applications of seed cells. As precursors of multiple neural cells, neural stem cells (NSCs) theoretically confer benefits over alternatives for SCI repair.^[^ ^48^, ^49^, ^50^, ^51^ ^]^ However, NSCs show a propensity to generate astrocytes at the expense of multilineage neural cells during neuroinflammation after SCI, leading to regeneration failure.^[^ ^52^, ^53^ ^]^ Additionally, the application of NSCs is expensive with a low yield. Hence, NSCs are far from meeting the requirements of clinical utility at a large scale. Here, we delivered hAESCs rather than NSCs to animal models to prepare them for further clinical applications. hAESCs are derived from placenta and are widely available and inexpensive with no ethical concerns. Based on their specific location in native tissue, hAESCs are regarded as a barrier between the fetus and mother and exhibit hypoimmunogenicity and no telomerase expression.^[^ ^30^, ^31^, ^54^, ^55^ ^]^ The immunoregulatory characteristics of hAESCs facilitate the improvement of a microenvironment around the lesion during the acute phase. hAESCs also express and secrete neurotrophic factors that facilitate nerve growth and extension. Therefore, hAESCs have great potential for the clinical translation of SCI repair.

Biomaterials are the backbone of engineered tissue scaffolds that support cell attachment and tissue regeneration.^[^ ^56^ ^]^ Similar to cells and other issues used in clinical practice, safety and hypoimmunogenicity are essential requirements for biomaterials as well.^[^ ^57^ ^]^ GelMA is a formable hydrogel that has been widely used for the repair and regeneration of various tissues with only few adverse reactions reported.^[^ ^58^, ^59^ ^]^ However, owing to its poor mechanical properties, GelMA has been mixed with some excipients to enhance its formability.^[^ ^60^, ^61^, ^62^ ^]^ To meet clinical requirements, we avoided supplementing any other materials and realized the accurate manufacturing of pure GelMA with complex structures by optimizing printability. Thus, we could lower the “barrier to entry” for clinical application using a simple composition.

Manufacturing is the process of combining cells with biomaterials to construct artificial tissues. For clinical use, the effect of this process on the cells should be minimal to maintain their viability and function. Cell‐laden bioprinting, which is a rapid and targeted 3D assembly of cells and materials, has gained considerable attention.^[^ ^63^ ^]^ But free radicals are inevitably generated and released due to UV light and some initiators during the special mode of GelMA photocuring.^[^ ^64^, ^65^ ^]^ As part of the bio‐ink, cells suffer from the changes and stresses throughout the printing process, which may cause damage to cell activity and viability as evidenced by Figure S6b (Supporting Information). However, cell viability in our case (≈25%) was relatively low compared to that in our previous work.^[^ ^66^ ^]^ This may be attributed to much harsher printing conditions, including increased light intensity and longer light exposure required by fine TPMS structure fabrication. In addition, hAESCs are a type of primary stem cells from the placental tissue, which are likely more sensitive and vulnerable to the printing process than other cell lines. Hence, we printed HES and subsequently seeded hAESCs instead of using cell‐laden printing to maintain the proper state and native characteristics of the cell grafts. Free radicals only existed when GelMA is exposed to ultraviolet light and are completely removed in the following operation.^[^ ^67^ ^]^ Therefore, the issues about cellular damage and viability could be improved in our HES system.

To date, numerous methods have been proposed to improve cell‐seeding efficiency on scaffolds, such as centrifugal spinning,^[^ ^68^ ^]^ electrospinning,^[^ ^69^, ^70^ ^]^ centrifugal force,^[^ ^71^ ^]^ micro‐pore structure,^[^ ^72^, ^73^, ^74^ ^]^ medium perfusion,^[^ ^75^ ^]^ and surface acoustic waves.^[^ ^76^ ^]^ However, the common cellular density is ≈10^2^ cells mm^−2^ or 10^5^–10^6^ cells cm^−3^, which is far from the initial cell density of native tissues.^[^ ^77^ ^]^ To achieve more efficient cell seeding on scaffolds, we developed SAA postprocessing based on a dual driving forces model and fabricated the HES. The cell density and cell loading number improved significantly on both the 2D hydrogel surface and 3D scaffold, reaching the level of 3 × 10^3^ cells mm^−2^ or 5 × 10^7^–10^8^ cells cm^−3^, respectively. Parallel to the enhanced cell‐loading capacity, the time spent on cell seeding was sharply reduced to ≈2 min in the HES system, which is a major step in efficient operation, considering the time spent of at least 30 min to 4 h in other post‐printing cellularization techniques.^[^ ^72^, ^73^, ^75^ ^]^ Efficient cell loading and operation protocols are highly relevant for translation of HES to clinical applications. Further studies are needed to overcome the current upper limit of cell loading and attempt to seed multiple cell types with different functions to meet the physiological requirements of tissues and organs.

Moreover, convenient and standardized operations are also important in practical using, especially for clinical applications. Complicated processes lead to high barriers of usage and increase the risk of instability in the therapeutic effects. Therefore, we divided the cell‐seeding process from scaffold manufacturing to meet the different requirements of cells and manufacturing from temporal and spatial perspectives. In HES–hAESCs system, cell seeding enables to finish automatically after immersing scaffolds into cell suspension due to the presence of dual driving forces. This one‐step method for cell seeding is user‐friendly and efficient to produce cell‐loaded scaffolds without introducing other factors such as centrifuging, perfusion and injection.^[^ ^68^, ^71^, ^75^ ^]^ Thus, our proposal not only further simplifies the operation, but also reduces the potential damage on cell grafts.

Standardized and consumable scaffold products, which are easy to store and transport, can be implemented by sterilization, lyophilization and modification. Additionally, standardized cell agents can be seeded on commercial scaffolds several hours or days in advance to construct artificial tissues according to the needs of the patients. Indeed, we have produced clinical‐grade hAESCs according to the Good Manufacturing Practice (GMP), whose safety and promising therapeutic effects have been proved in the clinical treatment of graft‐versus‐host disease (GVHD) (www.clinicaltrials.gov, #NCT03764228). In conclusion, this strategy is similar to the current clinical practice, with greater accessibility and stability, and could be suitable for clinical goals in the next decade.

Our approach was specifically designed to consider clinical requirements. However, several issues still need to be resolved prior to translation from bench to bedside, including safety concerns regarding methacrylic anhydride in GelMA and the initiator during photocross‐linking, and the fate of implanted cells in vivo. To this end, a clinical perspective is crucial to sensitizing research to real world needs and driving integration of new technologies into medicine.

## Conflict of Interest

The authors declare no conflict of interest.

## Author Contributions

C.Q. and Y.S. contributed equally to this work. L.Y., Y.H., C.Q., and Y.S. performed conceptualization; Y.S., C.Q., and J.L. performed methodology; Y.S., K.Y., G.W., and C.Q. performed in vitro bioprinting and manufacturing experiments; C.Q., Y.S., J.Z., J.L., and W.Y. performed in vitro cell culture and loading experiments; C.Q., Y.X., T.J., Y.K., and W.C. performed animal studies; C.Q., Y.S., Y.J., H.L. and Z.G. performed data analysis; C.Q., J.L., S.Z., Y.H. and L. Y. performed funding acquisition; C.Q and Y.S. wrote the original draft preparation; C.Q., Y.S., Z.H., Y.H., and L.Y. wrote, reviewed and edited.

## Supporting information

Supporting Information

Supplemental Video 1

Supplemental Video 2

Supplemental Video 3

Supplemental Video 4

## Data Availability

The data that support the findings of this study are available in the supplementary material of this article.
