# Supplementary material for: A 3D‐Printed Dual Driving Forces Scaffold with Self‐Promoted Cell Absorption for Spinal Cord Injury Repair
Source: Adv Sci (Weinh). 2023 Oct 23;10(33):2301639. doi: 10.1002/advs.202301639 (PMC10667844; doi:10.1002/advs.202301639)
Supplement: Supplementary file 1 — Supporting Information [file ADVS-10-2301639-s003.pdf]

ADVANCED SCIENCE
Open Access

Supporting Information

for *Adv. Sci.*, DOI 10.1002/advs.202301639

A 3D-Printed Dual Driving Forces Scaffold with Self-Promoted Cell Absorption for Spinal Cord Injury Repair

Chen Qiu, Yuan Sun, Jinying Li, Jiayi Zhou, Yuchen Xu, Cong Qiu, Kang Yu, Jia Liu, Yuanqing Jiang, Wenyu Cui, Guanghao Wang, He Liu, Weixin Yuan, Tuoying Jiang, Yaohui Kou, Zhen Ge, Zhiying He, Shaomin Zhang, Yong He and Luyang Yu**

Supporting Information

A 3D-Printed Dual Driving Forces Scaffold with Self-Promoted Cell Absorption for Spinal Cord Injury Repair

Chen Qiu†, Yuan Sun†, Jinying Li, Jiayi Zhou, Yuchen Xu, Cong Qiu, Kang Yu, Jia Liu, Yuanqing Jiang, Wenyu Cui, Guanghao Wang, He Liu, Weixin Yuan, Tuoying Jiang, Yaohui Kou, Zhen Ge, Zhiying He, Shaomin Zhang, Yong He, Luyang Yu**

The Supporting Information includes Experimental Methods, Supplemental Figures S1-S12, Supplemental Table S1, Supplemental Videos S1-S4 and Supplemental Data File S1 for all individual subject-level values.

Figure S1. Cellular distribution was influenced by gravity.
Figure S2. The contact angles of pure water on the dried sample surface.
Figure S3. SEM images of the sample surface.
Figure S4. The detection of attachment and growth of hAESCs on hydrogel sheets surfaces with different treatments.
Figure S5. Volume change and time consumption during swelling among dried, freeze-dried and SAA-treated scaffolds.
Figure S6. Cell viability in HES–hAESCs group and cell-laden printing group.
Figure S7. Upper limit of cell loading number of different HES scaffolds size.
Figure S8. Histological test of organs at 2 weeks and 8 weeks post-surgery.
Figure S9. Additional data about locomotor function *in vivo* at 8 weeks post-implantation.
Figure S10. Improvement of urinary system function at 8 weeks post-implantation.
Figure S11. Morphology and histology of spinal cord tissue around lesions.
Figure S12. Additional *in vivo* data of ultrastructure in lesions.
Table S1. Primers and probes for quantitative real-time PCR (RT-qPCR).
Data file S1. Individual subject-level values.
Video S1. hAESCs distribution after dropping onto scaffold.
Video S2. hAESCs distribution after injecting into scaffold.
Video S3. Free crawling of HES–hAESCs rats.
Video S4. Free crawling of FT rats.

1. Experimental Methods

1.1 Sample Collection and Experimental Design

Human amnion membranes were obtained with written and informed consent from healthy mothers undergoing cesarean section. The procedure was approved by the Institutional Patients and Ethics Committee of the Second Affiliated Hospital of Zhejiang University School of Medicine (project no. 2020-799). Written informed consent for research was obtained from participants prior to samples acquisition. And the informed consent confirmed that the participants voluntarily donated samples for research without any financial payment. hAESCs were isolated from the collected human amnion membranes for the investigations of HES-hAESCs *in vitro* and *in vivo*. To assess the therapeutic potential of HES–hAESCs for SCI treatment, animal experiments were conducted in the rat models with T10 full transection lesions. Rats were randomly divided into 4 groups: normal rats (normal), full transection rats

(FT), rats implanted with empty HES (HES) and with high hAESCs-loaded HES (HES–hAESCs). Female Sprague-Dawley rats (220-250g) were purchased from Slac Laboratory Animal Co. Ltd. (Shanghai, China). For animal experiments, work including feeding, rat models of SCI, a series of assessments of therapeutic effects, rat sacrificing and tissue harvesting, were performed strictly as the protocols approved by the Laboratory Animal Welfare and Ethics Committee of Zhejiang University (Ethics Code: ZJU20210074). Rats were housed in individual cages with free access to food and water and exposed to light on a 12-h cycle in a humidity- and temperature-controlled environment with no pathogenic microorganisms. We excluded the rats dying or presenting a sharply decrease in body weight post-operation. And the sample numbers of each individual experiment are present in the figure legends. All assessments of behavioral and histological tests were performed in a blinded manner. No criteria for inclusions and exclusions were set before the study. No samples or animals were excluded from analysis. The individual subject-level values are listed in data file S1.

1.2 Projection-based printing of TPMS scaffolds

The photocurable hydrogel ink was prepared by dissolving GelMA (EFL-GM-100-M1, Yongqinquan Intelligent Equipment Co., Ltd., Suzhou, China) in PBS at a concentration of 15% (w/v). Lithium phenyl-2,4,6-trimethylbenzoylphosphinate 0.5% (w/v) and tartrazine 0.05% (w/v) were added as photo-initiators and absorbers, respectively. The solution was stirred at 37°C for 20 min until fully dissolved and then loaded in to the ink tank of a projection-based bioprinter (EFL-BP-8601P, Yongqinquan Intelligent Equipment Co., Ltd., Suzhou, China). The TPMS model was selected from the device model library. Sixteen copies of the model were simultaneously printed at one time. We chose light intensity of 15 mW/cm^2 and 20 s exposure duration for each slice as the crosslinking parameters. The thickness of each layer was 50 µm. Printed scaffolds were first cleared in 37°C PBS twice, then disinfected and stored in 75% medical alcohol for further processing.

1.3 SAA postprocessing

Postprocessing was conducted in a sterile environment. The disinfected TPMS scaffolds mentioned in the previous step were cleaned in deionized water three times to remove alcohol and allowed to swell overnight. Then, the scaffolds were heated to dry in an oven at 60°C and their mass was measured until the mass of the samples was reduced to 34.8%, which corresponds to 75% water loss from hydrogel scaffolds via drying. Importantly, during this process, the scaffolds must be placed on a rotating bracket or turned over frequently to ensure the even removal of water. After that, the half-dried scaffolds were frozen at -80°C for 6 hours. Then, the scaffolds were further freeze-dried for 24 hours to remove the remaining 25% water.

1.4 Contact angle measurement

Flake samples (5 × 5 × 1 mm) were printed using the same materials and methods used for the TPMS scaffold. The flake samples were dried, SAA-processed, and freeze-dried. The contact angle between the pure water droplets and the samples was measured using a video-based contact angle measuring device (Dataphysics, Germany). 3 µL water droplet was formed by being extruded from a microsampler and touched the sample surface slowly and gently (pendent drop mode). A camera recorded the entire process as the droplet touched the surface until it was fully absorbed by the sample. The contact angle was measured at each moment from the recorded videos.

1.5 Mechanical properties test

The mechanical tests were conducted using an electronic universal testing machine (UTM2203; SHENZHENSUNS, Shenzhen, China). For the compression test, cylindrical samples with a

diameter of 5.28 mm and height of 4.932 mm were produced by projection-based printer. The loading rate was set to 0.2 mm/min. The compression strength was chosen as the largest stress before failure, whereas the compression modulus was calculated using the stress and strain within the elastic zone (5–10% strain). For the tensile tests, the actual stretched area was 5 × 5 × 1 mm. Dumbbell-shaped samples were produced by projection-based printer. The loading rate was set to 0.1mm/min. The stress-strain curve was automatically recorded using the testing system. The tensile strength was chosen as the largest stress before failure while the tensile modulus was calculated from the stress and strain of the entire curve (the stress-strain curve of tensile testing was approximately linear).

1.6 Pre-labelling of hAESCs

To trace implanted hAESCs *in vivo*, they were prelabeled with CellTracker CM-Dil (C7000, Invitrogen, USA) *in vitro*. Briefly, hAESCs was incubated in the basic DMEM media with 2 g/mL CM-Dil for 5–8 min in 37℃ and then transferred into 4℃ for 15 min. Next, hAESCs labeled with red fluorescence were used in further experiments.

1.7 Cell seeding into various 3D scaffolds

A total of 4-5 × 10^6 hAESCs were resuspend in about 200 μL DMEM basic media for cell seeding on different scaffolds (the cell number and media volume depend on the specific applications).

For HES, we used a standard and efficient method of cell seeding. First, the hAESCs suspension was added to 24 or 48 wells plate or an Eppendorf tube with a proper inner diameter for HES size (the inner diameter of the container is crucial for absorption efficiency of HES: too large or too small diameters decrease the absorption efficiency). HES was then thrown into the hAESCs suspension for approximately 2 min. During this time, HES rapidly and autonomously absorbed hAESCs into the scaffold.

For the NTS, dried, and freeze-dried scaffolds, the cell seeding process mentioned above is not suitable due to the lack of driving forces for cell absorption. Therefore, we chose a more common method for seeding hAESCs into these scaffolds. The scaffolds were first placed in the same container as in the HES seeding process, and then the hAESCs suspension was dropped onto top of the scaffolds. The timing of cell seeding was maintained for 2 min, which was the same as that for the HES.

After cell seeding and absorption, all scaffolds were carefully removed from the suspension and allowed to stand for approximately 1 h in a cell incubator to enable the hAESCs to attach. The hAESC-loaded scaffolds were cultured in culture medium until implantation.

1.8 Flow cytometry analysis for cell viability

For both seeding cell into the HES and cell-laden printing, the hAESCs density in the suspension or hydrogel was 1 million/mL. After seeding and printing, the hydrogel scaffolds were degraded using lysis solution to release cells, and hAESCs were collected by centrifugation. To detect cell viability, we used the Annexin V-FITC/PI kit (Yeasen, China) and performed flow cytometry (CytoFLEX S, Beckman, USA) according to the manufacturer's instructions. Live cells were labeled as Annexin V-FITC+ PI+, whereas late apoptotic and necrotic cells were labeled as Annexin V-FITC- PI-.

1.9 Evaluation of hAESCs proliferation on various 3D scaffolds

The CCK8 assay (Bergolin, Dalian, China) was performed *in vitro* to evaluate hAESCs proliferation seeded in the NTS, Dried, Freeze-dried and HES groups at 1, 3, and 7 days after cell seeding. The process of seeding cells is described above. All cell-loaded scaffolds (test groups) were cultured in the 24-well plate with 500 μL media, and those without cell-loading were regarded as blank groups cultured in the same condition. 50 μL CCK8 solution was added

into each well and then incubated in 37°C for 4 hours. After incubation, three 100-μl aliquots of each sample were transferred to a 96-well plate to perform three biological repeats. The optical density (OD) of each sample was measured at 450 nm to reflect the hAESCs proliferation on the NTS, Dried, Freeze-dried and HES 3D scaffolds.

1.10 Scanning electron microscopy (SEM)

SEM was used to observe the surface morphology of the GelMA scaffolds and the cell morphology of hAESCs on the scaffolds. Scaffold samples were fixed in 2.5% glutaraldehyde for 3-5 days, then in 1% osmium tetroxide. After dehydration in gradient ethanol, spinal cord samples were dried using a supercritical point dryer (HCP-2, HITACHI, Japan) and sputter-coated. The scaffolds were imaged using a scanning electron microscope (SU-8010; HITACHI, Japan) at the Analysis Center of Agrobiology and Environmental Science, Zhejiang University.

1.11 T10 full transection injury and post-surgical care

T10 transection was performed as described elsewhere *[13, 78]*. Briefly, the rats were deeply anesthetized and a midline incision was made over T10 to expose vertebral plate. Then rats underwent a T10 laminectomy and a transection of the spinal cord using a combination of micro-scissors and micro-stereoscope. A 2 mm spinal cord block was replaced with a 2.5-3 mm scaffold or 200 μL PBS buffer. Following implantation, the dorsal muscles and skin were sutured and antibiotics were administered. After surgery, rats were injected subcutaneously with 1 mL of saline and kept in a warm room until awakening. We manually emptied the rats' bladders twice a day and injected Baytril (1 mg/100 g) for 5 days to prevent urinary infection.

1.12 Behavioral assessments

The BBB open-field locomotion score was assessed weekly by investigators blinded to the treatment groups over an 8-week post-implantation period as previously described *[13, 42]*. The hindlimbs locomotion of each rat was assessed while free-walking in an open field and recorded as videos for 4 min, with the bladders emptied before testing. During the recording, the surrounding environment was maintained without stimulating the rats.

1.13 Upper thoracic spinal cord injection

Surgery for AAV2/9-EGFP injection was performed as previously described *[43]*. Briefly, the procedure before laminectomy was the same as that described above. AAV2/9-EGFP was injected into T7 spinal cord with the stereotaxic apparatus (69100, RWD, China), microinjection pump (LEGATO 130, KD Scientific, USA) and 10-μL Hamilton micro-syringe for anterograde tracing of propriospinal axons. Specific injection parameters were as follows: Injection speed: 100 nL/min; Injection sites: ± 0.8 mm lateral to the midline, three injection sites were separated by 1 mm and three different depths (0.5, 1.0 and 1.6 mm) for each site. The needle was left in place for 3 min before being moved to the next site. After injection, suture administration and post-surgical care were the same as those described in Section 1.9.

1.14 EMG recording and cortical stimulation

For EMG recordings while crawling freely in an open field, customized recording electrodes (AS632, Cooner Wire, USA) were wrapped to the mid-belly of the tibialis anterior (TA) muscle. The other end of the wire electrodes was connected to the recording head, secured on the skull under the skin. MEPs recordings of the cortical stimulation were conducted as previously described *[48]*. Briefly, the skulls of rat were exposed under anesthetized. The place of customized stimulation electrodes (762000, A-M System, USA) was in the motor cortex of left cerebral hemisphere, with the accurate position (x, y, z) is (2.95 mm, 1.65 mm, 2 mm) relative to Bregma (0, 0, 0). Recording electrodes were wrapped to the mid-belly of the right TA muscle, with the other end connecting with the Plexon (OmniPlex/128, USA). The single electrical

stimuli (5 mA constant current stimulus, 10 ms duration and 5 s intervals) was generated by pulse generator and isolator (A-M System, USA). EMG recordings were examined by a technician blinded to the treatment groups.

1.15 Histology and imaging

For immunofluorescence, the spinal cord tissues were fixed with paraformaldehyde (PFA) for 24 h and cryo-protected with 30% sucrose at 4℃ for 3 days. Then tissues were embedded with O.C.T. compound (4583, Sakura, Japan) in cryostat and sectioned in 30 μm. The sections were treated with 0.5% Triton-X-100 for 2 h, followed by a blocking solution containing 5% normal donkey serum and 5% bovine serum albumin at room temperature. Then the sections were incubated with primary antibodies at 4℃ overnight: Anti-GAP43 antibody (Millipore, AB5220); Anti-5-HT antibody (ImmunoStar, 20079); Anti-IBA1 antibody (Abcam, ab178847); Anti-CD68 antibody (Bio-Rad, MCA341GA); Anti-NF-H antibody (CST, 2836); Anti-NF-H antibody (Abcam, ab4680); Anti-NF-H antibody (Abcam, ab8135); Anti-Ki67 antibody (Abcam, ab15580). Secondary antibodies (room temperature for 1 hour) included: Donkey anti-mouse/rat/rabbit IgG secondary antibody, Alexa Fluor 488 (Invitrogen, R37114/A-21208/A-21206); Donkey anti-mouse/rat/rabbit IgG secondary antibody, Alexa Fluor 594 (Invitrogen, A-21203/A-21209/A-21207); Donkey anti-mouse/rat/rabbit IgG secondary antibody, Alexa Fluor 647 (Invitrogen, A-31571/A78947/A-31573); TRITC-conjugated donkey anti-chicken IgY (BBI, D110202); FITC-conjugated donkey anti-chicken IgY (BBI, D110201). Spinal cord section scan was imaged with Olympus VS200, and other transverse and horizontal sections were imaged with confocal microscopes (FV3000, Olympus, Japan).

For HE and other specific staining, the spinal cord tissues were fixed with paraformaldehyde (PFA) for more than 3 days, followed with embedded into paraffin and sectioned in 5 μm. Then the procedures of staining were followed by the manual of HE staining Kit (FD7451, FUDE Bio-tech, China) and Luxol Fast Blue staining Kit (G3242, Solarbio, China). Spinal cord section scan was imaged with Olympus VS200.

1.16 Transmission electron microscopy (TEM)

For assessing the synapse structure and myelination of axons in the lesions, the spinal cord tissues were prepared for TEM imaging. Rats were perfused with 4% paraformaldehyde and spinal cord tissue was fixed in 2.5% glutaraldehyde for 3-5 days, then fixed in the 1% osmium tetroxide and 2% uranyl acetate. After dehydration in a gradient of ethanol and 100% acetone, spinal cord samples were embedded in resin and sectioned by ultramicrotome, followed by imaging using a 100 kV microscope (Tecnai 10, Philips, Netherland) at Center of Cryo-Electron Microscope, Zhejiang University.

1.17 Statistical analysis

Statistical analysis was performed using GraphPad Prism 9. Student's t-test was used to compare means of two samples; One-way ANOVA with Tukey's post-hoc test was used to analyze the significance of differences between more than two samples. Data were presented as means ± SEM. P values < 0.05 were considered to be significant in statistics.

2. Supplementary Figures and Table

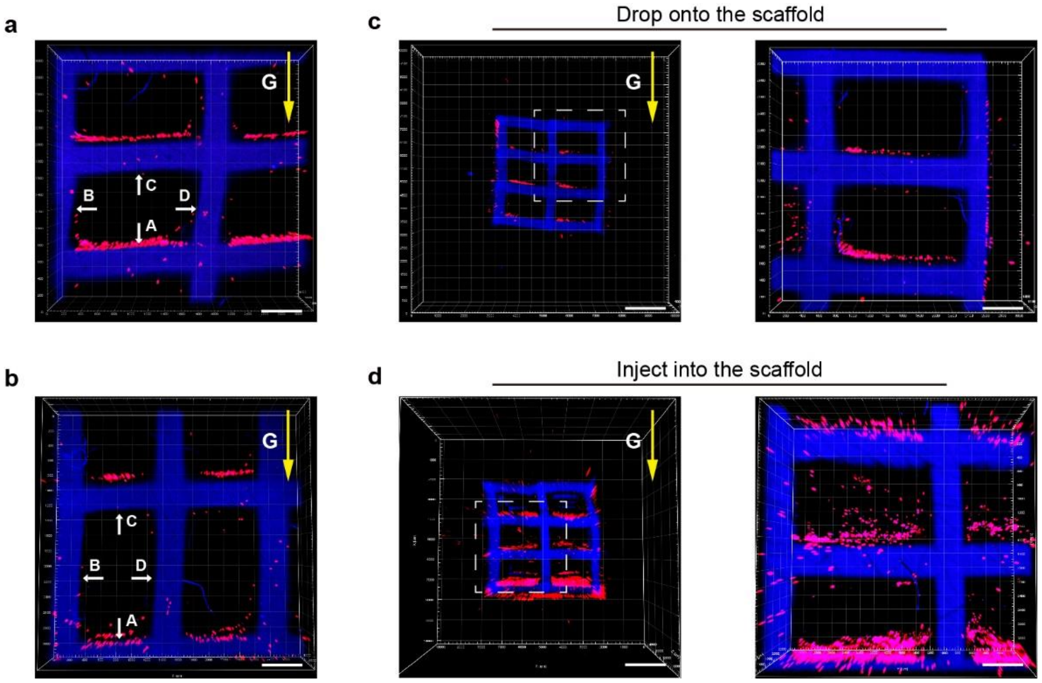

Figure S1. Cellular distribution was influenced by gravity (G, yellow arrows mean the gravity direction). The fluorescence images of hAESCs (red) being seeded on GelMA scaffolds (blue) after 12 hours (a time point for cell attachment and no exceeded proliferation). a-b) When cells were seeded on the regular hydrogel scaffolds, cells were prior to attach on surface A (white arrow) due to the gravity. Few cells attached on surface B, C and D, resulting in a low usage ratio of scaffold surface. Scale bar: 500 μm. c) The fluorescence images of hAESCs dropping onto the scaffold. Right side is the magnified view of white frame in left side. Scale bar: 1.5 mm, 500 μm. d) The fluorescence images when hAESCs were injected into the scaffold channels, right side is the magnified view of white frame in left side. Scale bar: 1.5 mm, 500 μm.

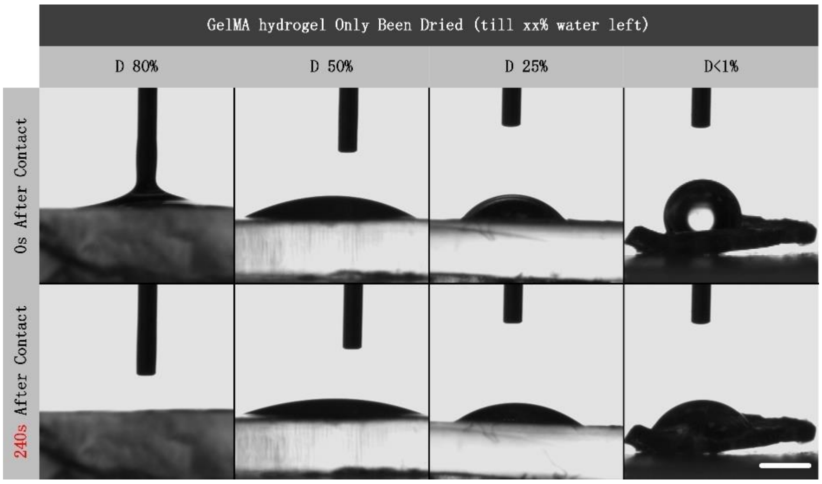

Figure S2. The contact angles of pure water on the dried sample surface. Hydrophilicity is positively correlated with water content. Water droplets can hold for more than 300 s on totally dried surface where water absorption occurs slowly. Scale bar: 500 μm.

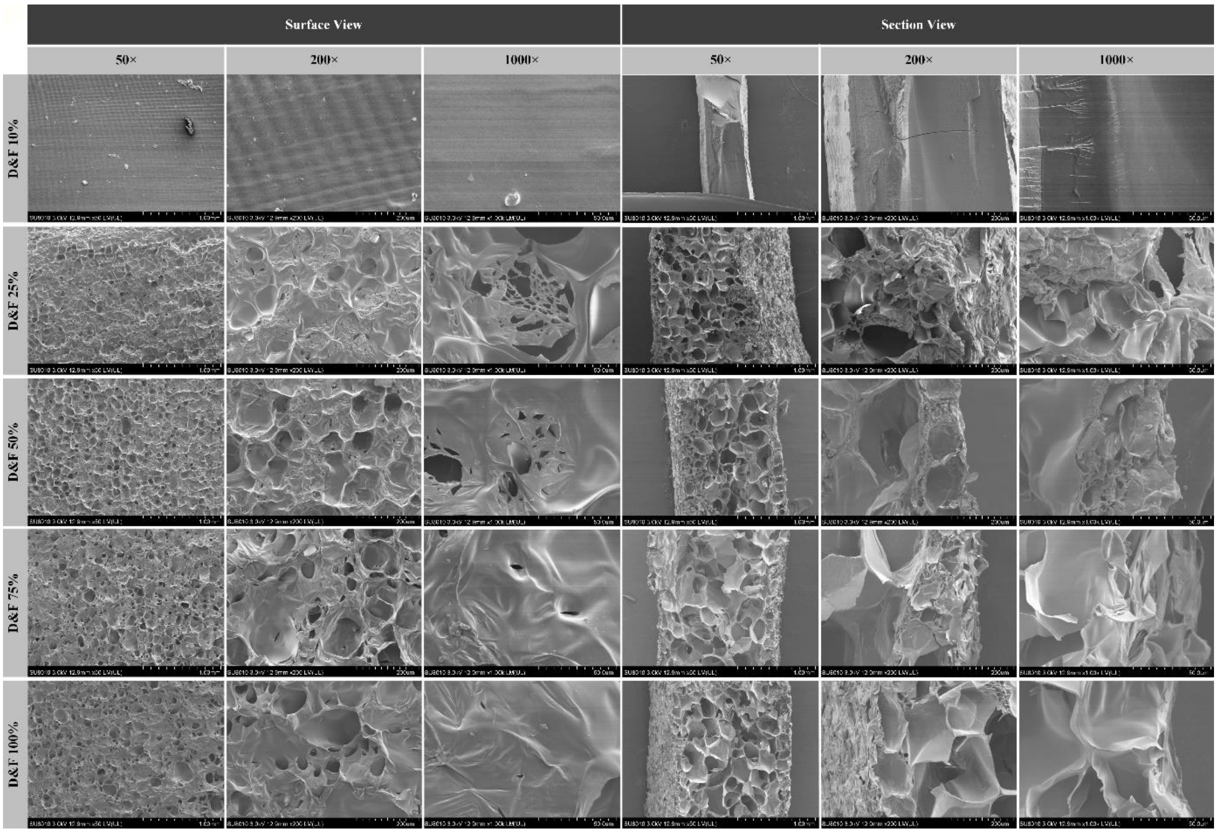

Figure S3. SEM images of the sample surface.

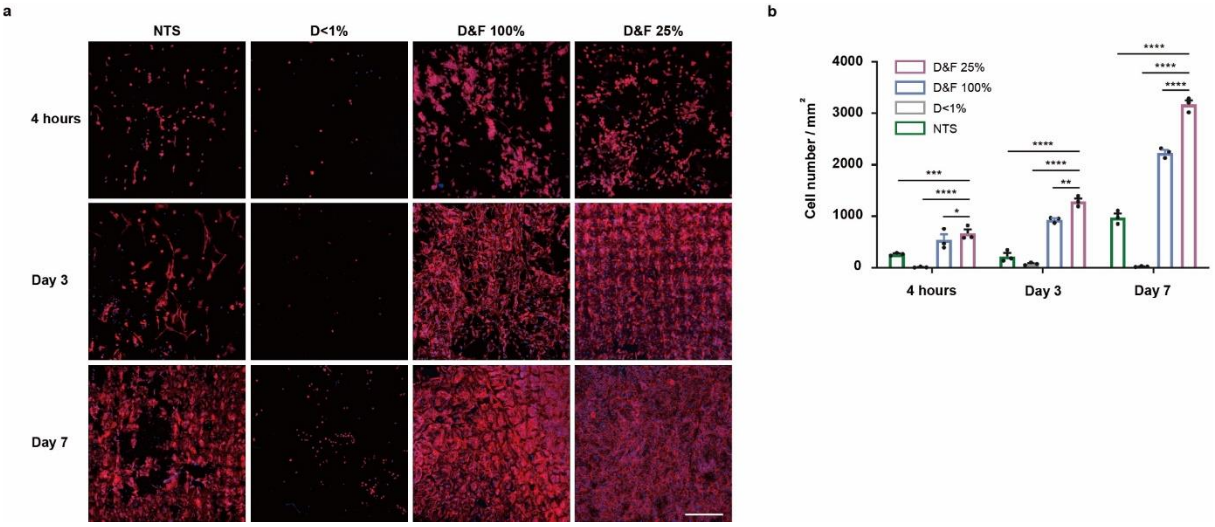

Figure S4. The detection of attachment and growth of hAESCs on hydrogel sheets surfaces with different treatments. a). Confocal images of hAESCs attaching and growing on hydrogel sheets surfaces with different treatments, cell skeleton was stained with red fluorescence (Phalloidin) and cell nucleus was stained with blue fluorescence (DAPI). Scale bar: 200 μm. b). Quantitative analysis of cell loading number on different hydrogel sheets surfaces. One-way ANOVA, followed by Tukey post-hoc test. n=3; ****$p < 0.0001$; ***$p < 0.001$; **$p < 0.01$; *$p < 0.05$; error bars, SEM.

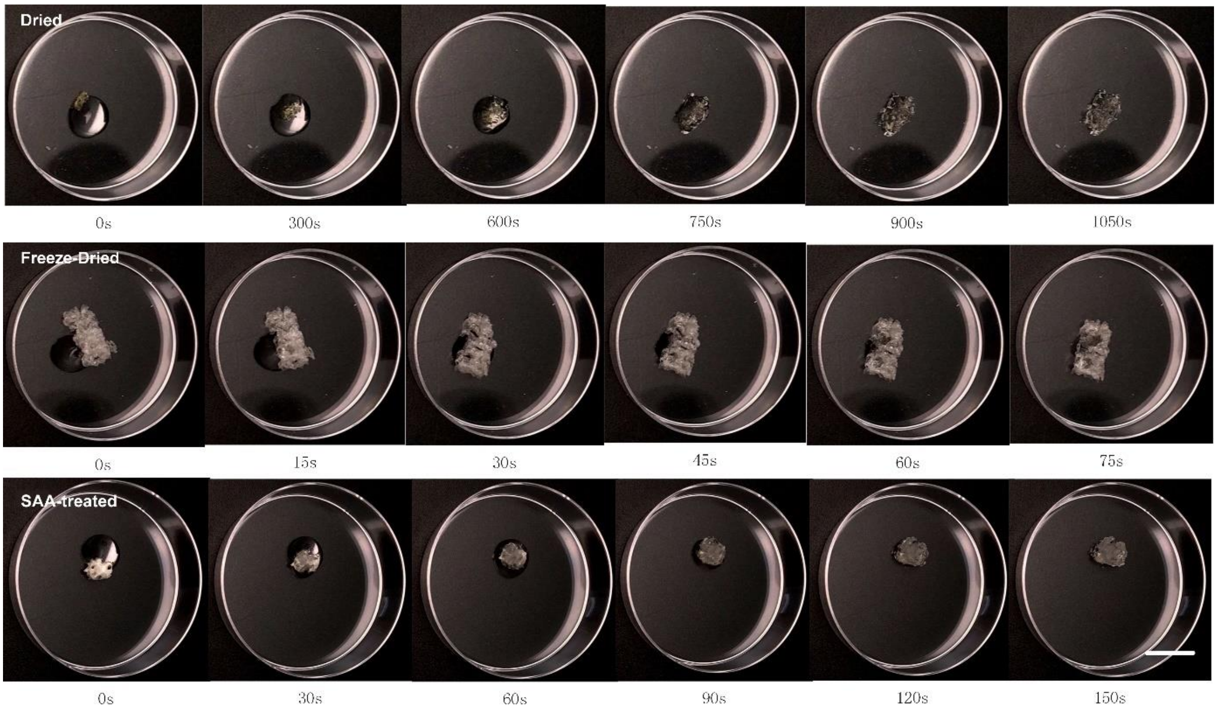

Figure S5. Volume change and time consumption during swelling among dried, freeze-dried and SAA-treated scaffolds.

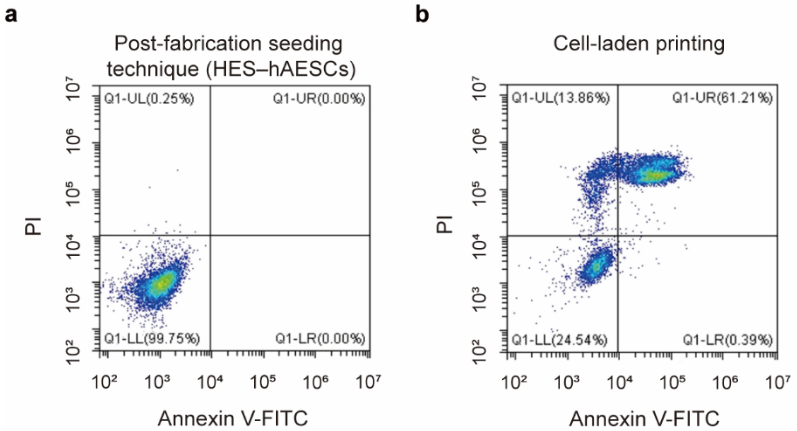

Figure S6. Cell viability in HES–hAESCs group and cell-laden printing group. Flow cytometry analysis of cell viability after seeding on HES (a) and cell-laden printing (b). a). More than 99% cells were viable after seeding on HES by post-fabrication seeding technique, showing by the Annexin V- and PI-. b). For cell-laden printing, only 24% cells maintain viability (Annexin V- and PI-), while more than 60% cells exhibited apoptotic or necrotic characteristics (Annexin V+ and PI+).

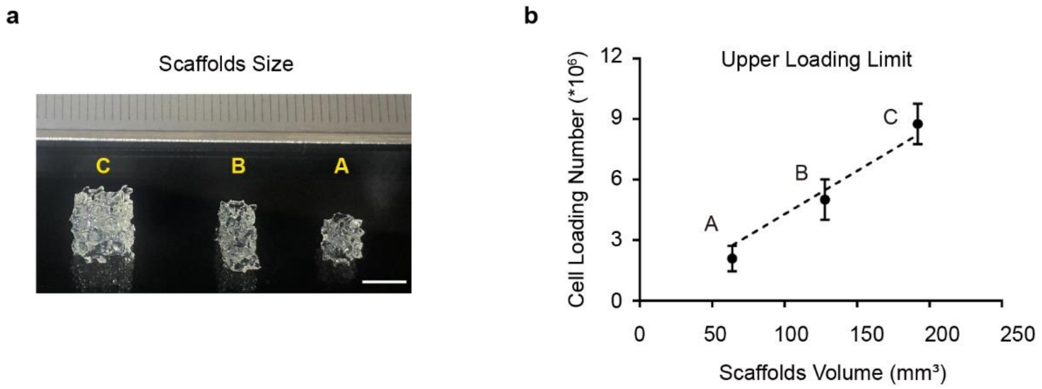

Figure S7. Upper limit of cell loading number of different HES scaffolds size. a) Representative images of HES scaffolds with different size. Scale bar: 5 mm. b) Upper loading limit of HES scaffolds with different size, error bars, SD.

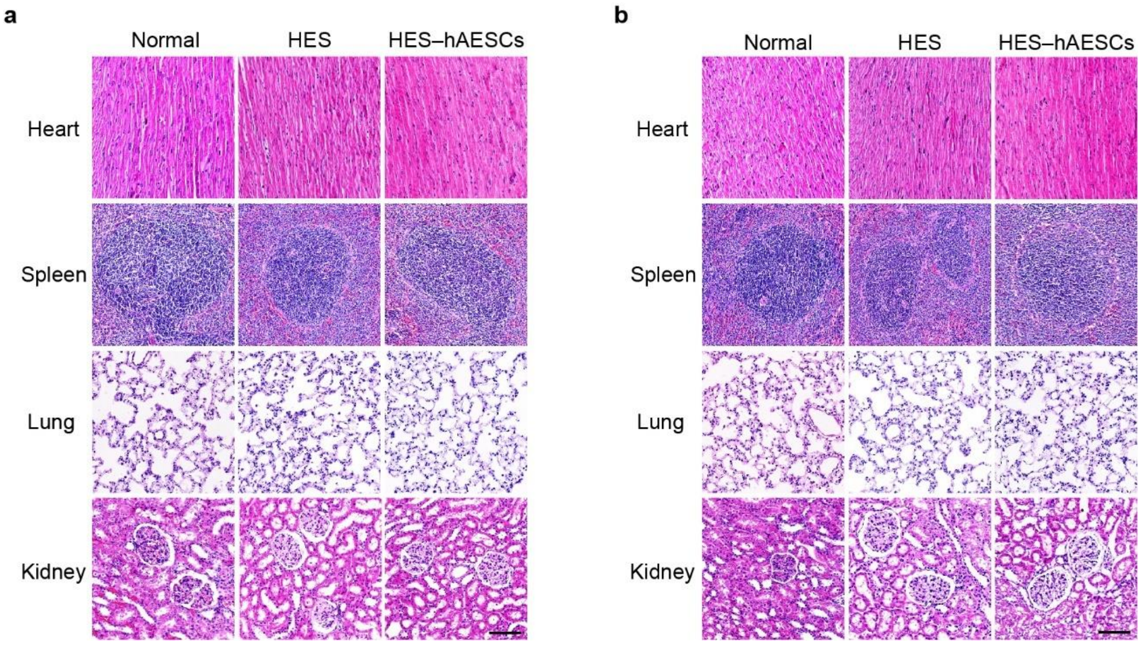

Figure S8. Histological test of organs at 2 weeks and 8 weeks post-surgery. a) HE staining of heart, spleen, lung and kidney at 2 weeks post-implantation. Scale bar: 100 µm. b) HE staining of heart, spleen, lung and kidney at 8 weeks post-implantation. Scale bar: 100 µm.

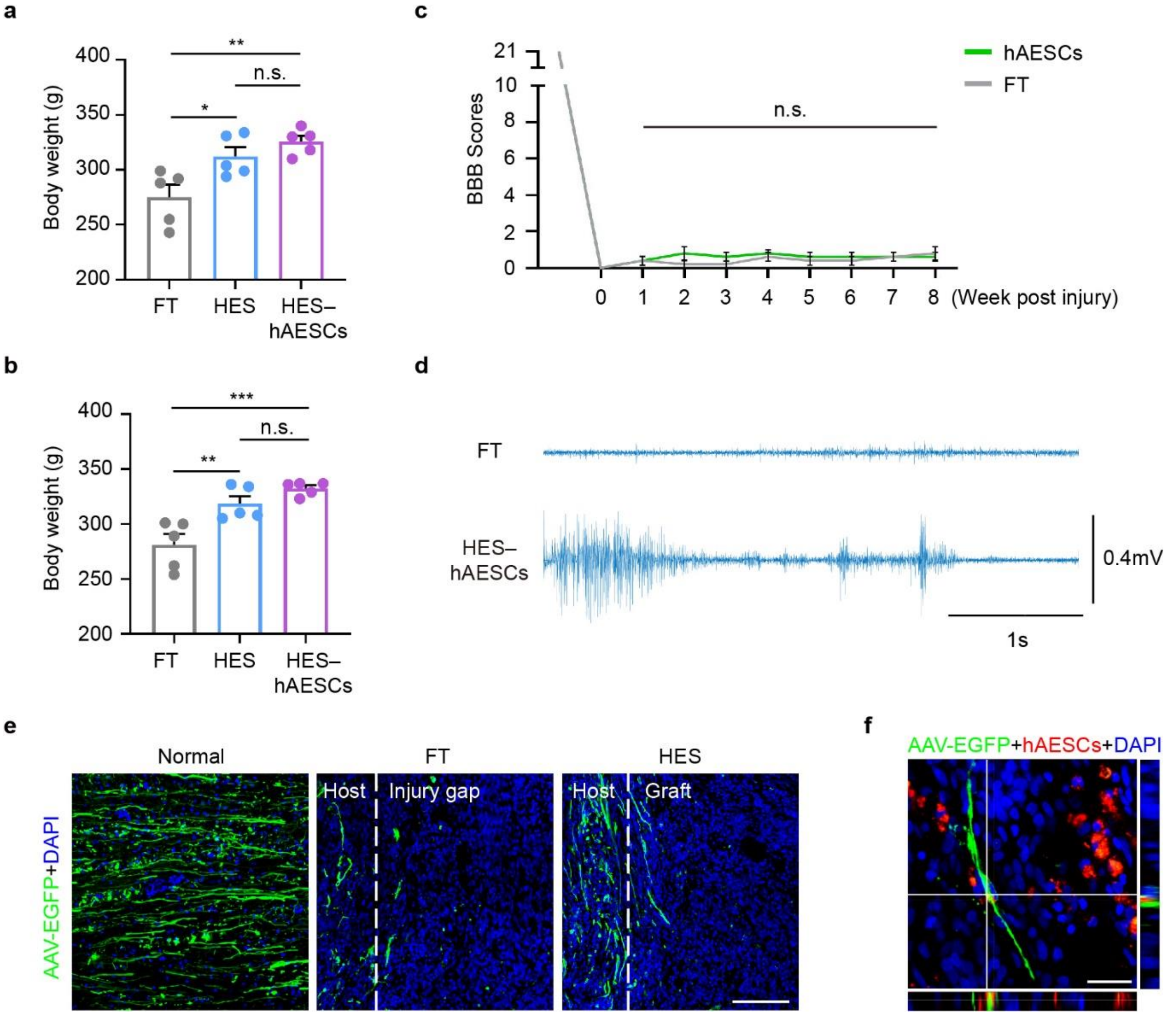

Figure S9. Additional data about locomotor function *in vivo* at 8 weeks post-implantation. a) Statistical analysis of body weight from indicated groups at 4 weeks post implant. One-way ANOVA, followed by Tukey post-hoc test. n=5; ***p < 0.001; **p < 0.01; *p < 0.05; n.s., not significant; error bars, SEM. b) Statistical analysis of body weight at 8 weeks post-implant. One-way ANOVA, followed by Tukey post-hoc test. n = 5; ***p < 0.001; **p < 0.01; n.s., not significant; error bars, SEM. c) BBB locomotion scores of hAESCs-treated rats and FT rats in a 8-week period. Student' s t-test. n=5; n.s., not significant. d) Representative EMG images from indicated groups while crawling freely in open field at 8 weeks post-implant. e) Representative images of AAV-EGFP labeled descending axons from indicated groups. White dotted lines showed the interface between host spinal cord and injury gap (in FT rats) or HES graft (in empty HES-treated rats). Scale bar: 100 µm. f) Descending EGFP axons partly converge on the hAESCs (red fluorescence), forming a kind of nourishing structure. Scale bar: 25 µm.

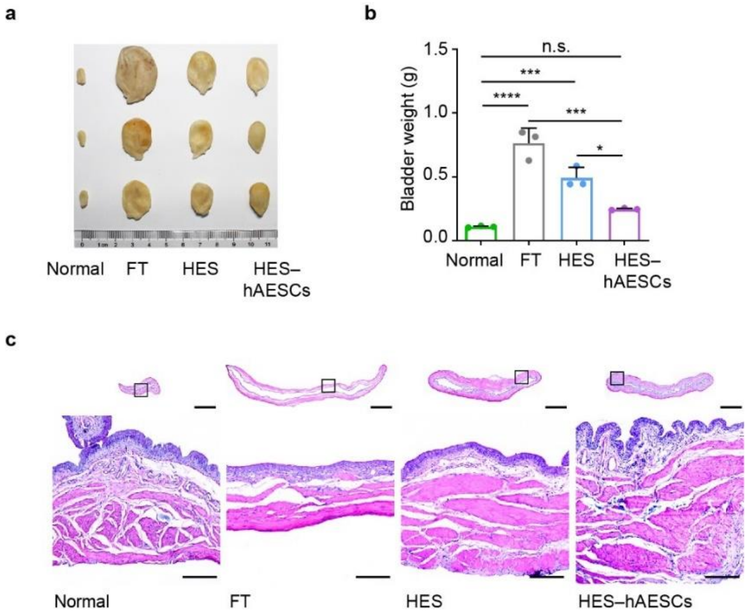

Figure S10. Improvement of urinary system function at 8 weeks post-implantation. a) The images of whole bladders from indicated groups. b) Statistical analysis of bladder weight from indicated groups. One-way ANOVA, followed by Tukey post-hoc test. n=3; ****p < 0.0001; ***p < 0.001; *p < 0.05; n.s., not significant; error bars, SEM. c) HE staining showed the histological structure of the bladders from indicated groups. Scale bars: 1 mm, 200 μm.

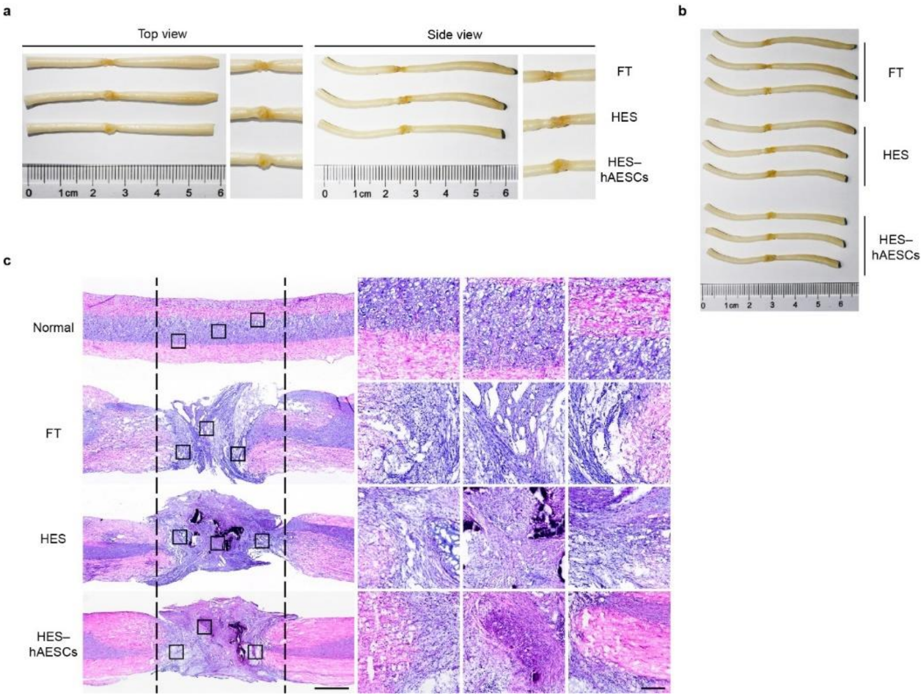

Figure S11. Morphology and histology of spinal cord tissue around lesions. a-b) Morphological display of spinal cord tissue around lesions from indicated groups. Magnified views of epicenter showed apparently atrophic symptom in FT rats. c) HE staining of spinal cord around lesions from indicated groups. The part of right side showed the magnified images from black frames in left images respectively. Scale bars: 1 mm, 200 μm.

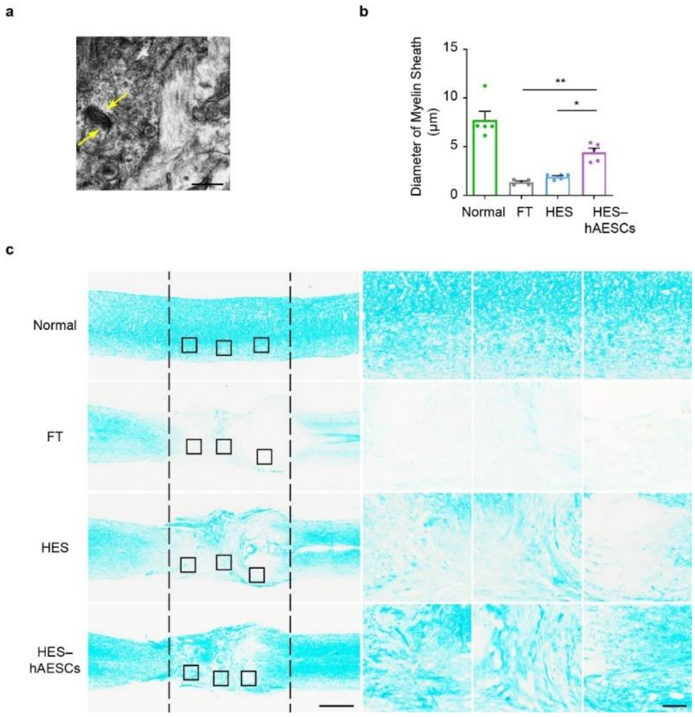

Figure S12. Additional *in vivo* data of ultrastructure in lesions. a) Yellow arrows showed the excitatory synapses with rounded vesicles in scaffold channels loaded with hAESCs. Scale bar: 500 nm. b) Quantitative analysis of the diameter of myelin sheath in the lesions from indicated groups. One-way ANOVA, followed by Tukey post-hoc test. n = 5; $^{**}p < 0.01$; $^{*}p < 0.05$; error bars, SEM. c) Luxol fast blue staining showed the degree of myelination around the lesions from indicated groups. Right side showed the magnified images from black frames in left side. Scale bars: 1 mm, 200 μm.

Table S1. Primers and probes for quantitative real-time PCR (RT-qPCR)

Primer and probe	Sequence
rat GAPDH-F	AAGGTCGGTGTGAACGGATTTG
rat GAPDH-R	TGTAGTTGAGGTCAATGAAGGGGTC
rat CD68-F	GAGCCAGAGCAAGGATTT
rat CD68-R	CATTCGCTTCAAGGACATA
rat IL-6-F	GATTGTATGAACAGCGATGATGC
rat IL-6-R	AGAAACGGAACTCCAGAAGACC
rat IL-1β-F	CCCAACTGGTACATCAGCACCTCTC
rat IL-1β-R	CTATGTCCCGACCATTGCTG
rat IL-10-F	GACAACATACTGCTGACAGATTCCT
rat IL-10-R	TCACCTGCTCCACTGCCTTG
rat BDNF-F	TTAGCGAGTGGGTCACAGCGG
rat BDNF-R	CGAGTTCCAGTGCCTTTTGTCTATG

F: Forward; R: Reverse

References:

[78] B. Chen, Y. Li, B. Yu, Z. Zhang, B. Brommer, P. R. Williams, Y. Liu, S. V. Hegarty, S. Zhou, J. Zhu, H. Guo, Y. Lu, Y. Zhang, X. Gu, Z. He, *CELL* **2018**, *174*, 521.
